# Molecular Characterization of Acquired Tolerance of Tumor Cells to Picropodophyllin (PPP)

**DOI:** 10.1371/journal.pone.0014757

**Published:** 2011-03-14

**Authors:** Jamileh Hashemi, Claire Worrall, Daiana Vasilcanu, Mårten Fryknäs, Luqman Sulaiman, Mohsen Karimi, Wen-Hui Weng, Weng-Onn Lui, Christina Rudduck, Magnus Axelson, Helena Jernberg-Wiklund, Leonard Girnita, Olle Larsson, Catharina Larsson

**Affiliations:** 1 Department of Molecular Medicine and Surgery, Center for Molecular Medicine, Karolinska Institutet, Karolinska University Hospital, CMM L8:01, Stockholm, Sweden; 2 Department of Oncology-Pathology, Karolinska Institutet, Karolinska University Hospital, CCK R8:04, Stockholm, Sweden; 3 Department of Genetics and Pathology, Rudbeck Laboratory, Uppsala University, Uppsala, Sweden; Cairo University, Egypt

## Abstract

**Background:**

Picropodophyllin (PPP) is a promising novel anti-neoplastic agent that efficiently kills tumor cells *in vitro* and causes tumor regression and increased survival *in vivo*. We have previously reported that PPP treatment induced moderate tolerance in two out of 10 cell lines only, and here report the acquired genomic and expression alterations associated with PPP selection over 1.5 years of treatment.

**Methodology/Principal Findings:**

Copy number alterations monitored using metaphase and array-based comparative genomic hybridization analyses revealed largely overlapping alterations in parental and maximally tolerant cells. Gain/ amplification of the *MYC* and *PVT1* loci in 8q24.21 were verified on the chromosome level. Abnormalities observed in connection to PPP treatment included regular gains and losses, as well as homozygous losses in 10q24.1-q24.2 and 12p12.3-p13.2 in one of the lines and amplification at 5q11.2 in the other. Abnormalities observed in both tolerant derivatives include amplification/gain of 5q11.2, gain of 11q12.1-q14.3 and gain of 13q33.3-qter. Using Nexus software analysis we combined the array-CGH data with data from gene expression profilings and identified genes that were altered in both inputs. A subset of genes identified as downregulated (*ALDH1A3*, *ANXA1*, *TLR4* and *RAB5A*) or upregulated (*COX6A1*, *NFIX*, *ME1*, *MAPK* and *TAP2*) were validated by siRNA in the tolerant or parental cells to alter sensitivity to PPP and confirmed to alter sensitivity to PPP in further cell lines.

**Conclusions:**

Long-term PPP selection lead to altered gene expression in PPP tolerant cells with increase as well as decrease of genes involved in cell death such as *PTEN* and *BCL2*. In addition, acquired genomic copy number alterations were observed that were often reflected by altered mRNA expression levels for genes in the same regions.

## Introduction

Resistance to cancer treatment is a major cause of disease recurrence and mortality in modern oncology. On the functional level, drug resistance may affect processes such as uptake and processing of the drug, modifications of the target and induction of DNA repair [1]. Alterations related to the cell cycle, programmed cell death and escape of apoptosis are other important factors in therapy resistance. Underlying molecular alterations can be found at the DNA level, e.g. copy number variation and sequence mutation, at the transcriptional level e.g. regulatory or posttranscriptional changes or involve translational or posttranslational modification of proteins. Epigenetic modifications have also been shown to affect drug sensitivity and resistance. These include e.g. altered methylation of individual gene promoters [2], [3], [4] or genome-wide changes [5], as well as alterations in expression of microRNAs [6].

The cyclolignan picropodophyllin (PPP) is a promising anti-cancer agent with documented tumor inhibitory effects. In experimental systems, PPP causes massive apoptosis of malignant cells, reduced cell motility *in vitro* and *in vivo* tumor regression was observed not only in xenografted mice and mouse tumor models [7]-[13] but also in intracerebral rat xenografts [14]. Furthermore, PPP at therapeutically achievable doses prolonged survival by up to three months in mouse models of multiple myeloma [9]. The drug was well tolerated by the animals and in addition to the reduction of tumor burden, decreased tumor-associated angiogenesis and osteolysis were observed [9], [15], [16]. PPP has been reported to cause ubiquitination of the insulin like-growth factor 1 receptor (IGF-1R) and thereby inhibit its activation, which caused attenuation of the phosphatidyl-3 inositol (PI3K) anti-apoptotic pathway [7], [8], [12], [13], [17]-[19]. However, PPP did not inhibit the highly homologous insulin receptor, thereby circumventing the risk for diabetogenic responses associated with many other anti-IGF-1R therapies. An oral IGF-1R inhibitor (AXL1717) of cyclolignan chemistry is presently studied in cancer patients (phase I/II) with successful results (www.axelar.se).

Our previous selection experiments on cultured tumor cells failed to produce resistance to PPP [7]. PPP treatment produced only weak tolerance in two out of ten tumor cell lines selected for increasing doses of PPP over an 80-week period [7]. Moreover, the multidrug resistance proteins MDR1 and MRP1 were not found to be affected by long-term PPP treatment [7], consistent with another study showing that PPP effectively killed multidrug resistant osteosarcoma cells [12].

Even though *in vitro* studies have suggested low incidence for PPP resistance, it is important to explore the potential genetic mechanisms underlying tolerance to PPP, not least from a clinical perspective as these types of compounds may in future be used in treatment of human cancer. In this context we have here evaluated genome-wide gene expression patterns and copy number genomic alterations in association with *in vitro* long term PPP treatment in human cancer cells.

## Materials and Methods

### Cell lines and culturing

The generation of PPP tolerant derivatives from the established human cancer cell lines Line2 and Line3 have been previously reported [7]. The parental cell line Line2, corresponding to DFB melanoma cells originally named DFBmel [20], was kindly provided by Prof. Rolf Kiessling at Karolinska Institutet, and Line3, ES1 Ewing sarcoma cells, was obtained from American Type Culture Collection (Rockville, MD) [8]. The parental cell lines were selected by incubation in increasing concentrations of PPP as described in [7]. The IGF-1R expression, IGF-1R dependency and PPP responsiveness of the parental cells has been previously reported [7]. To generate PPP tolerant derivatives, Line2 and Line3 cells were exposed to increasing doses of PPP from 10 to 500 nM over an 80-week period resulting in derivatives with different levels of PPP tolerance, as previously reported [7]. As paired control cell lines, the parental untreated cells were cultured in parallel for 80 weeks. The suffix 500 and 200 in Line2-T500 and Line3-T200 refers to the highest levels of PPP tolerance obtained, in nM. The parental and tolerant cells used in the present study were stored at −135 ^°^C, thawed and cultured for 30 days in the absence or presence of PPP at the same concentration as the highest observed tolerance.

The BE melanoma cell line was a kind gift from Prof. Rolf Kiessling at Karolinska Institutet, and MCF7 breast cancer was obtained from American Type Culture Collection (Rockville, MD) [8]. BE and MCF cell lines are PPP non-tolerant and were used as controls in siRNA and cell viability experiments.

### DNA extraction

Genomic DNA was isolated from cultured cells according to standard procedures applying either phenol/ chloroform purification and ethanol precipitation, or using a commercial kit (QiagenGmbh, Hilden, Germany). DNA samples were subsequently used for genotyping, metaphase comparative genomic hybridisation (CGH), array-CGH and methylation analysis.

### Genotyping of parental and PPP tolerant cells

Short tandem repeat (STR) profiling was performed on all cell lines assayed by metaphase-CGH using the AmpF*l*STR Profiler Plus ^TM^ PCR amplification kit and an ABI 377 DNA sequencer (Applied Biosystems, Foster City, CA, USA). The markers included D3S1358, FGA, D5S818, D7S820, D8S1179, vWA, D13S317, D18S851, D21S11 and Amelogenin. Parental and PPP treated Line2 cell lines at all tolerance levels tested (40, 100, 300 and 400 nM) were characterized by the genotypes 15; 20; 11/12; 9/11; 11/13; 16/17; 11/12; 15; 28/31; and X/Y. Parental and PPP treated Line3 cells (40 and 100nMand 200 nM at two different time-points) showed the genotypes 15; 24; 13; 8/9; 13/14; 16; 12; 13; 30; and X.

In addition, genotyping of single nucleotide polymorphism (SNP) was carried out at the Mutational Analysis Facility (MAF), Karolinska Institutet, Stockholm, for all lines analysed by array-CGH using the marker panel and analysis system introduced by Hannelius *et al* [21]. In total, 47 SNPs were genotyped using a Sequenom^TM^ mass spectrometer and results were compared pair-wise between parental and maximally tolerant cell lines.

### Spectral karyotyping (SKY)

SKY analysis was performed as described [22] on metaphase spreads of parental Line2 and Line3 cells. Image acquisitions were performed using a SD200 Spectracube system (ASI) mounted on a Zeiss Axioskop microscope (Carl Zeiss Jena GmbH, Jena, Germany) with a custom-designed optical filter (SKY-1, Chroma Technology, Brattleboro, VT, USA). Karyotypes were based on analyses of 7 and 10 complete chromosome spreads for Line2 and Line3, respectively. Descriptions of karyotypes follow the recommendations of the International System for Human Cytogenetic Nomenclature (ISCN 1995) [23].

### Metaphase-CGH

CGH was performed on parental and PPP tolerant Line2 cells (40, 100, 300, 400 and 500 nM) and Line3 cells (40 and100 nM and 200 nM PPP at two different time-points) using methods previously described [24]. DNA was extracted from ∼6.0×10^6^cells in monolayer cultures. In brief, 1 µg cell line DNA (test) was labelled with FITC (green, NEN Life Science, Boston MA, USA) and the normal DNA (reference) with Spectrum Red (red, Vysis, Inc.), mixed with human COT-1 DNA (Invitrogen) and hybridized together onto denatured metaphase spreads from normal male lymphocytes (Vysis, Inc.). Images were captured with a Zeiss Axioplan 2 and analyzed with the Isis/CGH software (Metasystems, Germany). Based on green-to-red ratios, copy number alterations were classified as losses (ratios≤0.8), gains (≥1.2) or amplifications (≥1.5). Heterochromatic regions, the short arms of the acrocentric chromosomes and chromosomes X and Y were excluded and GC rich regions in 1p, 19 and 22 were interpreted with caution. As controls, normal male DNA was hybridized against normal female DNA and cell lines were hybridized and analyzed on two separate occasions.

### Array-CGH

Array-CGH was performed on parental and tolerant cells at the maximum tolerated PPP level including Line2, Line2-T500, Line3 and Line3-T200. Generation of microarrays, experimental procedures and data analyses have previously been described in detail [25]. The tiling arrays applied were produced from a 33,370 BAC clone library (CHORI BACPAC resources, (http://bacpac.chori.org/genomicRearray.php) at the SCIBLU Genomics Centre, Lund University, Sweden (www.lu.se/sciblu) providing a resolution of one clone per 50–100 kb.

One microgram of cell-line test DNA and normal reference DNA (Promega, USA) were labelled separately with Cy3-dCTP and Cy5-dCTP (PA53021, PA55021; GE Healthcare) using a random labelling kit (BioPrime Array CGH Genomic kit; Invitrogen Life Technologies, Carlsbad, CA, USA), purified with a Purelink PCR purification kit (Invitrogen Life Technologies, Carlsbad, CA, USA) and co-precipitated with human COT-1 DNA (Invitrogen). DNA probes were subsequently resuspended in 50 µl of formamide-based hybridization solution, denatured and hybridized to arrays in chambers (Corning Inc, Corning, NY) for 72 h at 37 ^°^C. After hybridization and stringent washing (50% formamide/2×SSC at 45 ^°^C for 20 min, 2×SSC/0.1% SDS at 45 ^°^C for 30 min and 0.2×SSC at room temperature for 15 min), slides were dried by nitrogen blowing and scanned using a Genepix 4200A confocal scanner (Axon instruments Inc., Union City, CA).

Images were quantified using the GenePix Pro 6.0 package analysis software (Axon instruments, Wheatherford TX, USA) and uploaded at the BioArray Software Environment (BASE; http://www.base.thep.lu.se/) [26] for filtering, normalization and statistical analysis, as described in [27]. First, spots flagged by the GenePix software or disqualified after manual inspection were removed. Subsequently, probes with spot diameter <40 µm or intensity below background were removed. Background-corrected data was obtained by calculating the median foreground and subtracting the median background signal intensity for each channel. Intensity ratios for individual probes were calculated within arrays from background-corrected intensity levels of test sample divided by reference sample and data were normalized using the pin-based LOWESS algorithm [28]. The data was smoothed using a three-clone sliding window and CGH plotter [29] was used to identify the extent of alterations using a segmentation constant of 7. Log_2_ ratios for two continuous BAC clones outside the threshold ranges were classified as copy number changes including gain (threshold +0.25), loss (−0.25), amplification (+1) and homozygous loss (−1). Small aberrations in the telomeric regions were not taken into account due to chromosomal instability in these regions.

Information about physical and cytogenetic locations of clones was according to the UCSC genome browser (http:/www.genome.ucsc.edu/; July 2004 freeze). Our findings from the 33k array were compared with a previous analysis using a commercially available 1 Mb resolution array (Spectral Genomics, Houston, TX USA) and showed concordant results (data not shown).

### Fluorescence *in situ* hybridization (FISH)

FISH analysis was performed on Line2, Line2-T500, Line3 and Line3-T200 cells using a commercially available Spectrum Orange labelled probe for the *MYC* locus at 8q24.21 (Vysis LSI C-MYC Abbott Molecular, Illinois, USA). The results were analyzed in a Zeiss Axioplan 2 epifluorescence microscope (Carl Zeiss Jena GmbH, Jena, Germany) and documented using a SD200 Spectracube system (ASI). For each cell line, between 30 and 60 interphase nuclei were scored for the number of *MYC* signals with additional evaluation of 2–5 metaphases.

### Gene expression profiling

RNA was prepared from Line2, Line2-T500, Line3, and Line3-T200 cells using the RNeasy miniprep kit (Qiagen, Chatsworth, CA) and used for expression analysis, which was carried out at the Uppsala Array Platform. Gene expression profiling was performed using the Genome U133 Plus 2.0 arrays with methodology as recommended in the GeneChip Expression Analysis Technical Manual (Rev. 5, Affymetrix Inc., Santa Clara, CA). Raw data was processed by MAS5 in the Affymetrix GeneChip Operating Software (GCOS) to generate quantitative signals and qualitative Detection Calls.

### Integration of array-CGH and expression data by Nexus

The array-CGH data was processed in the Nexus 3.1 (BioDiscovery Inc., El Segundo, CA, USA) software using the built-in Rank segmentation algorithm to identify regions of relative copy-number alterations in Line2-T500 vs. Line2 and Line3-T200 vs. Line3 cells. The following parameters that were different from default were applied: p = 1×10^−6^, a minimum of 5 probes, and ±0.2 as a threshold for gain or loss. The identified regions were subsequently integrated with corresponding Affymetrix expression data, with separate analyses of alterations in Line2-T500, Line3-T200, and Line2-T500 and Line3-T200 combined.

### Identification of dysregulated genes in tolerant lines and functional annotations

Differentially expressed genes between Line2-T500 vs. Line2 and Line3-T200 vs. Line3 cells were identified using two different criteria. Gene list U was generated by MAS5 including genes with >2-fold difference between parental and PPP treated cells and giving a “present” detection flag in both cell lines (Uppsala Array Platform).

For gene list H, more stringent criteria were used (at core facility BEA, Huddinge, Karolinska Institutet). This time probes were selected based on the following criteria i) increased or decreased call (significant difference in expression), ii) minimum of a two-fold change in expression levels (Signal Log Ratio (SLR)≥1 and SLR≤−1 respectively) and iii) Present Call in the samples. The 193 probe sets from gene list H identified as commonly dysregulated in both lines (78 up-regulated and 115 down-regulated) were entered into hierarchical clustering using the R software programme (http://www.r-project.org/). Functional annotation was performed for gene list U and H using the database for annotation, visualization and integrated discovery (DAVID) (http://david.abcc.ncifcrf.gov/) [30]. Functional classification categories with enrichment scores ≥1and p-values <0.05 were studied in more detail.

### Treatment with siRNA and assessment of cell viability

Gene specific Silencer Select siRNA (Ambion) against six genes downregulated in PPP tolerant cell lines (*ALDH1A3*, *ANXA1*, *TLR4*, *SPIN3*, *SOCS3* and *RAB5A*) were transfected into non-tolerant Line2, Line3, BE and MCF7 cells. Silencer Select siRNA against seven genes upregulated in PPP tolerant cell lines (*COX6A1*, *LGALS2*, *NFIX*, *ME1*, *BCL2*, *MAPK* and *TAP2*) were transfected into Line2-T500, Line3-T200 PPP tolerant and non-tolerant, BE and MCF7 cells. Sequences for siRNA are available on request.

Transfection of siRNA was performed with Lipofectamine RNAiMAX (Invitrogen) at a ratio of 10 pmol siRNA to 0.6 µl RNAiMAX. A reverse transfection mixture using 6 pmol siRNA per well was plated into wells of a 96 well plate and 5,000 cells/well added. Untransfected cells, RNAiMAX only and negative siRNA controls were included for each cell line. The cells were incubated for 24 h and the medium changed to 100 µl fresh medium with and without 0.5 µM PPP. After a further 48 h, 10 µl of alamarBlue cell viability reagent (Invitrogen) was added, the cells incubated for 4 h at 37 °C and the fluorescence emission at 585 nm measured (Tecan Infinite M1000). It was confirmed that the fluorescence measurements were within the range for which they correlate linearly with cell number. The sensitivity to PPP was assessed by calculating the ratio between the cell viability measurement of siRNA treated cells with and without PPP treatment, to normalize for effect on cell viability of the siRNA transfection procedure. For the sensitivity curves, Line2 untransfected and transfected with *ALDH1A3* or *ANXA1*siRNA and Line2-T500 untransfected or transfected with *COX6A1* and *NFIX* siRNA in 96 well plates were incubated after 24 h with 0, 0.05, 0.5, 1 and 2.5 µM PPP for 48 h and cell viability measured with alamarBlue.

### qRT-PCR to confirm effect of siRNA on gene expression

Line2 and Line2-T500 were transfected with siRNA using Lipofectamine RNAiMAX in the same ratios as for the functional experiments except in 6 well plates and after 36 hours, the RNA extracted using the GeneJET RNA purification kit (Fermentas) according to the manufacturer's instructions. RNA concentration was determined by NanoDrop spectrophotometer and 1 µg was incubated in 1×DNase buffer with 1 U of DNase I for 30 mins at 37 °C in a total volume of 10 µl. The solution was brought to 5 mM EDTA and heated to 65 °C for 10 mins to inactivate the enzyme. RTase buffer, 0.5 µg oligo dT primer, 1 mM dNTPs and 200 U of RevertAid reverse transcriptase (Fermentas) were added to the DNase treated RNA to a total volume of 20 µl and reverse transcribed into cDNA through incubation at 50 °C for 30 mins followed by enzyme inactivation at 85 °C for 5 mins. Primers for quantitative real-time reverse transcription polymerase chain reaction (qRT-PCR) were designed using the Primer 3 programme (http://frodo.wi.mit.edu/primer3/) and were synthesised by Sigma Aldrich (Table S1). Primers were dissolved in TE buffer at 100 µM and then diluted to a working concentration of 20 µM with water. The PCR reaction mixture consisted of 1×Maxima SYBR Green/ROX qPCR Master Mix (Fermentas), 1 µl cDNA and 0.3 µM of both forward and reverse primers in a total volume of 10 µl. Ten fold serial dilutions of cDNA from the untreated cell lines, from 1 to 1:100, was analysed and used to construct a standard curve for each gene. For each primer pair a negative control PCR reaction was performed that did not contain cDNA template. The qRT-PCR reactions were assembled in MicroAmp 384 well optical reaction plates (Applied Biosciences). Each gene and cDNA combination was analysed in triplicate. The plates were sealed with a lid and centrifuged for 1 min at 5,000 rpm. The reaction plates were placed in an ABI Prism 7900HT sequence detection system (Applied Biosystems) and heated to 50 °C for 2 min, 95 °C for 10 min to activate the polymerase and denature the primers followed by 40 cycles of 60 °C for 1 min annealing, extension occurring during increase to 95 °C and 95 °C for 15 s denaturation. Fluorescence was measured and data collected at 95 °C and 60 °C during each cycle. Following completion of the amplification, products were denatured by heating from 60 °C to 95 °C, during which data was collected to produce dissociation curves. The data was analysed by the SDS 2.3 programme. The threshold was automatically selected at a point where the increase in fluorescence was exponential. The SDS 2.3 produces a standard curve for each pair of primers and calculates the quantity for each unknown sample from the line of best fit from the standard curve and calculates the quantity mean for the 3 well replicates. It was confirmed that the dissociation curves showed a single peak at the correct melting temperature for the primer pair (Table S1). The quantity mean for each test gene was divided by the GAPDH quantity mean for that sample to adjust for the amount of cDNA present.

### Luminometric methylation assay (LUMA)

Genome-wide DNA methylation at CCGG sites was determined by LUMA in the parental Line2 and Line3 and PPP tolerant Line2-T500 and Line3-T200 cells. For methylation assay, the parental Line2 and tolerant Line2-T500 cells were cultured for 24 h with 500 nM PPP (total PPP for tolerant Line2-T500 was 1000 nM). The PPP dose for parental Line3 and tolerant Line3-T200 was 200 nM (total PPP for Line3-T200 was 400 nM).

The LUMA methodology has been described in detail elsewhere [31]. In short, each cell line DNA sample was digested with *Hpa*II and *Eco*R1 or *Msp*I and *Eco*R1 followed by cleavage quantification using Pyrosequencing in a PSQ96 MA system (Biotage AB, Uppsala, Sweden). Peak heights were determined with the PSQ MA software for calculation of *Hpa*II/*Msp*I ratios (*Hpa*II/*Eco*R1)/(*Msp*I/*Eco*R1). A *Hpa*II/*Msp*I ratio approaching one indicates 0% methylation while a ratio approaching zero suggest 100% methylation. All experiments were performed twice on separate occasions.

## Results

### Genotyping of parental and PPP tolerant cell lines

STR profiling demonstrated identical genotypes between the parental Line2 and the treated Line2 cell lines at the different levels of tolerance, as well as between Line3 and the tolerant Line3 cell lines. High concordance in SNP genotypes was also observed between Line2 and Line2-T500 (91%), and between Line3 and Line3-T200 (91%).

### Characterization of parental cell lines by SKY

The chromosomal compositions of parental Line2 and Line3 cells were characterized by SKY (Figure S1). Line2 displayed a hypertriploid chromosome content and Line3 was found to be hyperdiploid. Although most of the breakpoints observed in Line2 and Line3 were clearly different, some commonly involved regions could be identified. Specifically, regions 2p22, 5q21, and 21p11 that contain known fragile sites, were found rearranged in both lines, however each involved different translocation partners (Figure S1). The results were in agreement with our previous exclusion of gross rearrangement at the *IGF1R* locus in 15q26.3 using FISH analyses with flanking genomic clones [7].

### Metaphase CGH abnormalities during PPP treatment

The chromosomal gains and losses identified by metaphase CGH in parental and tolerant cell lines are illustrated in Figure S2. Gains, losses and amplifications involving a subset of chromosomes were identified in all parental and tolerant cell lines. Amplifications were identified in chromosomal regions 3p11-p14 and 7q32-qter in parental and PPP treated Line2. Alterations of chromosome 11 were observed as the only common aberrations. Losses involving 11q23-qter were detected in parental and PPP treated Line2 at 40 nM and higher concentrations and gains in 11p12-q13 were acquired in Line2-T100 and cell lines tolerant to higher concentrations of PPP. Furthermore, while Line3 cells did not exhibit alterations of chromosome 11, gain of 11p12-q13 and loss of 11q23-qter both occurred from Line3-T100 onwards. Thus, common to both cell lines were gains in 11p12-q13 that appeared at 100 nM and persisted until the end of study. Other alterations observed in PPP treated cell lines include gain of 13q22-qter and 17p11.2-p12 in Line3 T (Figure S2).

### Array-CGH abnormalities in PPP tolerant cell lines and verification by FISH

Array-CGH was performed on parental and maximally PPP tolerant cell lines of Line2 and Line3 using tiling BAC arrays. Copy number alterations related to PPP treatment were identified by comparison between abnormalities in parental and PPP tolerant cell lines. These changes are of interest since they may represent resistance mechanisms. In addition to the scoring of copy number alterations using CGH plotter, log_2_ ratio profiles were compared manually between parental and tolerant cell lines, which identified a few distinct alterations with ratios at but not outside the thresholds, and these were considered as borderline alterations. Furthermore, given that PPP tolerance could only be achieved in a minority of cell lines, abnormalities present in parental cell lines were also considered as possibly contributing to the PPP resistance.

**Figure 1:**
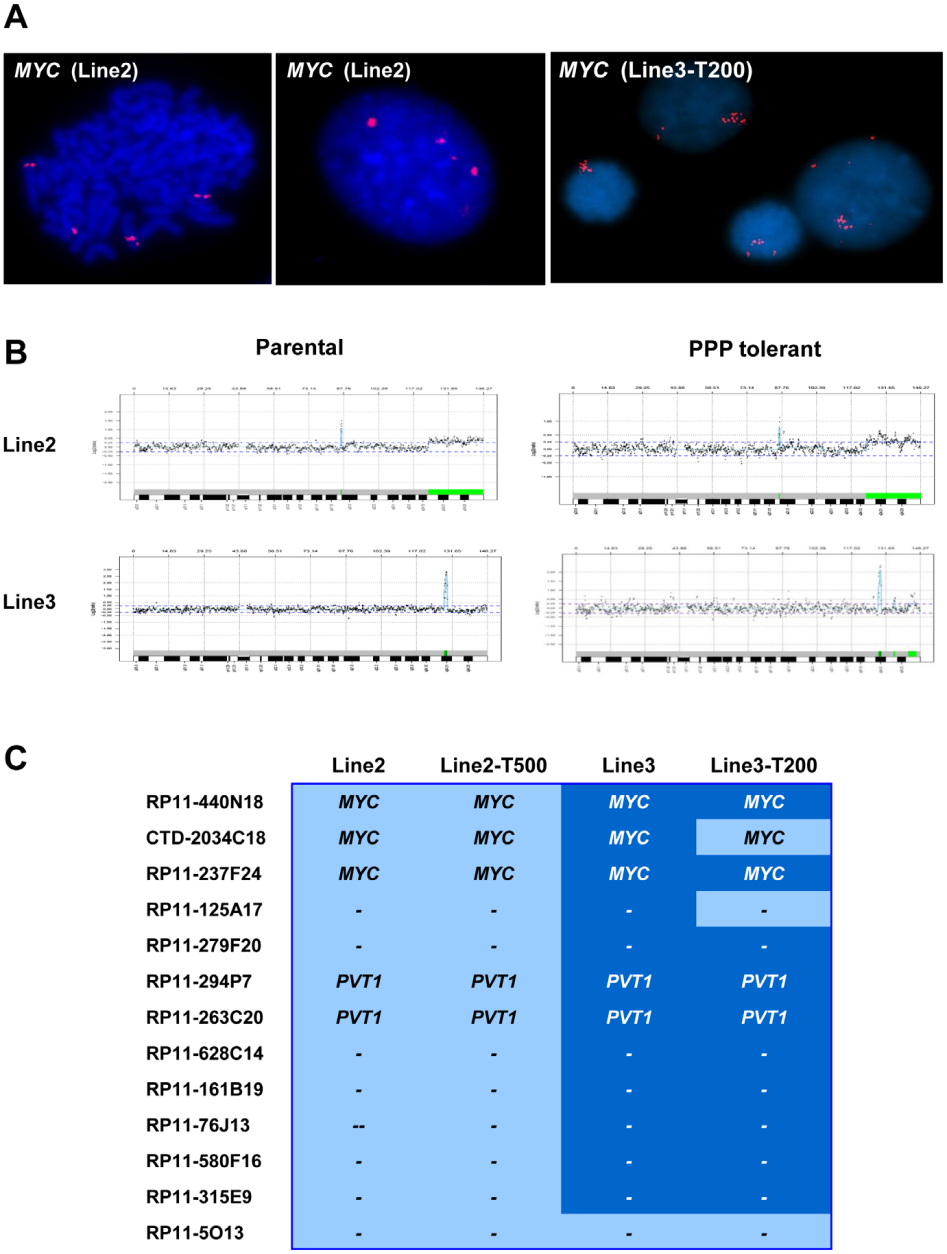
Gains and amplifications involving 8q24.21 and verification of array-CGH results by FISH for the *MYC* locus. (**A**) FISH analysis using a Spectrum Orange labelled BAC clone covering the *MYC* locus. Line2 cells in metaphase show red signals on 4 individual chromosomes as well as 4 signals in interphase. In interphase nuclei of Line3-T200 cells single individual red spots are observed together with clusters of more than 10 signals suggesting a homogeneously staining region. (**B**) Array-CGH profiles for chromosome 8 in parental and PPP tolerant cells. Parental and PPP tolerant cells show gain of 8q24.13-qter in Line2, narrow amplification of 8q24.21 in Line3. (**C**) The heatmap illustrates the common regions of gain/amplification including the *MYC* and *PVT1* loci, with gain indicated in light blue and amplifications marked in dark blue. The first column is the name of clones in the BAC array.

The detected copy-number abnormalities are detailed for each cell line in Tables S2 and S3. Almost all chromosomes were affected in both parental and tolerant cell lines. In addition to regular gains and losses, amplifications and homozygous losses were also observed (Tables S2 and S3). Among these can be noted the homozygous loss including the *CDKN2A* locus at 9p21.3 in Line2 and Line2-T500. Copy number gains or amplifications involving 8q24.21 were detected in all lines (Figure 1) and were verified by locus specific FISH analysis for the *MYC* locus (Figure 1). At array-CGH Line2 and Line2-T500 revealed similar gains of 8q24.13-qter. FISH analyses detected mean signal numbers of 3.5 (range 2–6; n = 56) in Line2 and of 3.4 (2–6; n = 57) in Line2-T500, with single signals observed on different chromosomes in metaphase. In Line3 and Line3-T200 cell lines, array-CGH showed narrow amplifications at 8q24.21. In these lines FISH analyses showed mean numbers of 7.2 (range 4–12; n = 31) in Line3 and of 5.1 (3–10; n = 42) in Line3-T200, and in metaphases the majority of signals were found clustered on one chromosome suggesting a small homogenously staining region (HSR, Figure 1). Detailed analysis of the gained interval in 8q24.21 showed that *MYC* and *PVT1* were both gained or amplified in parental and PPP tolerant cell lines.

Parental and PPP tolerant cell lines were identical in the majority of copy number aberrations detected. Consequently, only a limited number of acquired gains and losses in relation to PPP tolerance were identified (Table 1). In addition, some alterations identified in parental cell lines were not retained in the tolerant derivatives (Table S1). Alterations of larger chromosome segments were found in tolerant cells including losses (5p, 6, 9, 12p and 21q) and gains (5q, 10, 11, 12p, 13q and 18q) in Line2-T500, as well as losses (3p, 7q, 11q, 16q and 17q) and gains (1q, 3q, 4p, 5p, 8q, 11, 17q, 18q and 22q) in Line3-T200 (Table 1). Homozygous losses include 10q24.1-24.2 and 12p12.3-13.2 in Line2-T500, and amplification was found at 5q11.2 in Line3-T200 (Table 1).

**Table 1:** Copy number abnormalities observed by array-CGH in PPP tolerant cell lines only.

Chr	Line2-T500	Line3-T200
***Losses***		
3	-	3p12.3-13
5	5p14.3-pter	-
6	6p21.31-qter	-
7	-	7q35
9	9p11.3-q12	-
10	***10q24.1-q24.2 hz***	-
11	-	11q21-22.1; q22.1-qter
12	12p13.31-ter; ***p12.3-13.2 hz***	-
16	-	16q23.3-qter
17	-	17q23.2
21	21q11.2-22.3	-
***Gains***		
1	-	1q21.1-25.3; q32.1-3; q43-qter
3	-	3q27.3-qter
4	-	4p15.2-pter
5	(5q11.2)	5p15.1-pter; p13.3-q11.2/**q11.2**
8	-	8q24.22; q24.3
10	10p12.32-pter; q22.2-23.2	-
11	(11p11.12-q14.3)	11p11.12-12; q12.1-21
12	12p12.1-12.3	-
13	13q31.3-qter	13q33.3-qter Pr.
17	-	17q11.2; q12
18	18q21.2-21.31; q22.3-ter	18q12.1
20	20q13.2-13.31	-
22	-	22q12.3; q13.32-q13.33

Bold indicates amplification; Homozygous deletions hz are marked in bold and italic.

Borderline alterations are given within paranthesis; "Pr" indicates Pronounced gain.

**Figure 2:**
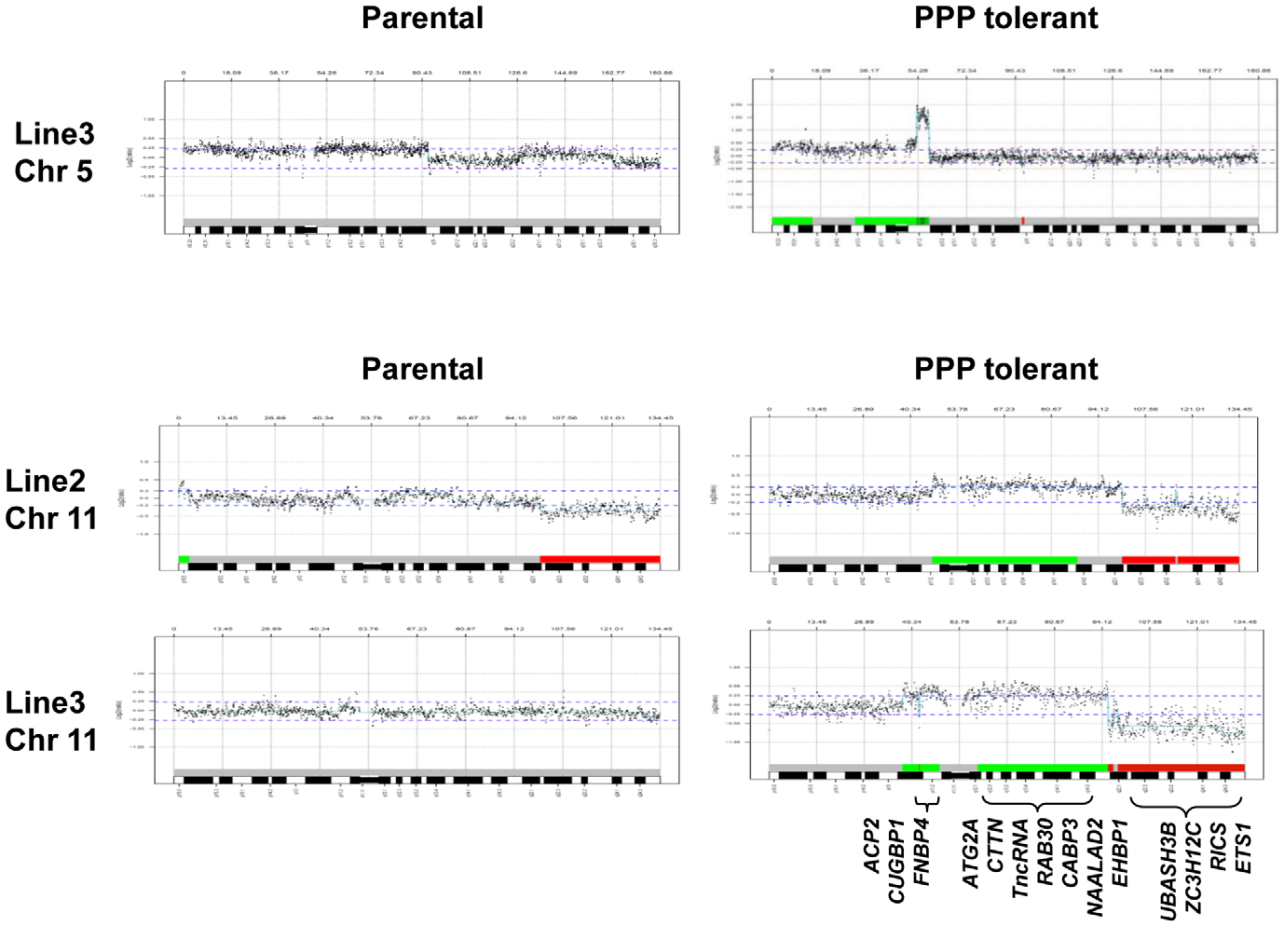
Array-CGH profiles of copy number alterations. Amplification of 5q11.2 in Line3-T200 cells (top) and chromosome 11 copy number alterations in all lines (bottom). Gain of 11q12.1-q14.3 is identified as a common alteration in Line2-T500 and Line3-T200 cells as compared to Line2 and Line3 cells. For Line2 and Line2-T500 the threshold +0.2 is used to illustrate the border-line gain of 11p11.2-q14.3. Copy number loss of distal 11q is present in Line3-T200 and in Line2 and Line2-T500. Candidate genes with common over-expression in the region of gain are shown below the plots. The array-CGH plots of chromosome 5 show alterations outside the thresholds of +0.25 for gain and +1.0 for amplification, as well as borderline alterations at the +0.25 threshold. The alterations were scored as borderline gain of 5pter-q15 in Line3, and in Line 3-T200 as gain of 5p15.1-pter and p13.3-q11.2 with borderline gain of p13.3-p15.1 and amplification of q11.2.

Although most sites of changes were different between Line2-T500 and Line3-T200, some commonly involved regions were identified such as 5q11.2, 11q12.1-q14.3 and 13q33.3-qter. The most prominent was the narrow amplification in 5q11.2 in Line3-T200 (Figure 2) and present as a borderline gain in Line2-T500 (Table 1). Increased copy number involving 11q12.1-q14.3 was detected as a gain in Line3-T200 and as a borderline gain in Line2-T500 (Figure 2). Gain of 13q31.3-qter was observed in Line2-T500 and the gain in chromosome 13 observed in Line3 was strongly pronounced for the interval 13q33.3-qter in Line3-T200 cells. Loss involving distal 11q was observed for 11q22.1-qter in Line2/Line2-T500 (Figure 2).

### Copy number imbalances in relation to gene expression levels in PPP tolerant cell lines

**Figure 3:**
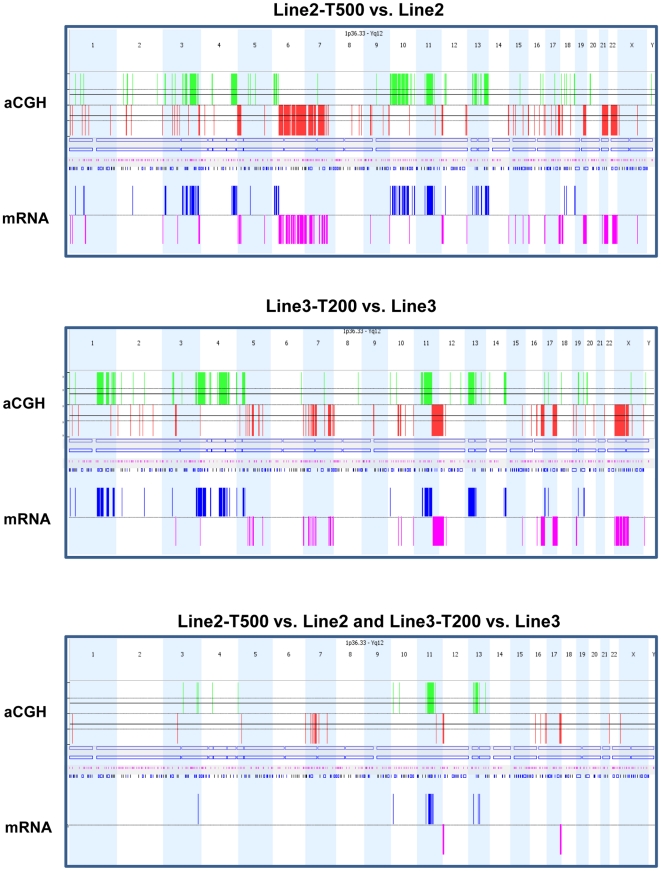
Comparison of copy number imbalances with corresponding gene expression levels. Comparison of relative copy number imbalances between tolerant and parental cells detected in Line2-T500 (top), Line3-T200 (middle), and commonly for Line2-T500 and Line3-T200 (bottom), with corresponding gene expression levels by Affymetrix. Each vertical line represents one abnormality in one sample. In each panel the upper plots for array-CGH data show relative copy number gains (green) and losses (red) from 1pter to Yqter. The lower plots show genes with increased (blue) or decreased (purple) expression at loci with altered gene dose in Line2 and Line3.

We next asked whether copy number variations between parental and PPP treated cell lines were reflected at the mRNA level as up-regulated or down-regulated genes. For this purpose, expression profiling was carried out for Line2, Line2-T500, Line3 and Line3-T200 using the Affymetrix platform. Relative copy number variations by array-CGH were identified by direct comparison of PPP-tolerant cell lines with parental cell lines using Nexus, and for all involved loci data were subsequently integrated with expression levels by Nexus. As illustrated in Figure 3, relative copy number increases were commonly associated with over-expression and copy number decreases with under-expression. Copy number imbalances detected in both Line2-T500 and Line3-T200 included relative gains in 3q, 4q, 10p, 11q and 13 and relative losses in 1p, 3p, 5p, 7p, 11q, 12p, 16p, 17q and 21q (Figure 3). Thirty-one genes with corresponding alterations in mRNA levels were identified including 23 over-expressed genes within 3q26.33-q27.1, 10p14, 11p11.2-p11.12, 11q11-q14.3, 13q12.3-q13.2, 13q13.3-q21.1 and 13q21.2 and 8 under-expressed genes within 12p13.33-p13.31 and 17q24.3-q25.3 (Table 2).

**Table 2:** Commonly dysregulated genes in regions of relative copy number alterations in PPP tolerant Line2-T500 and Line3-T200 cells (data by Nexus analyses).

Gene symbol		Location		Locus
	Gene name	Chr.	start-end (Mb)	Link ID
***Upregulated genes within regions of relative copy number gain***				
*FXR1*	fragile X mental retardation, autosomal homolog 1	3	182.1–182.3	8087
*CUGBP2*	CUG triplet repeat, RNA binding protein 2	10	11.1–11.5	10659
*ACP2*	acid phosphatase 2, lysosomal	11	42.2–42.3	53
*CUGBP1*	CUG triplet repeat, RNA binding protein 1	11	47.4–47.6	10658
*FNBP4*	formin binding protein 4	11	47.7–47.8	23360
*PATL1*	protein associated with topoisomerase II homolog 1	11	59.1–59.2	219988
*NAT11*	N-acetyltransferase 11 (GCN5-related, putative)	11	63.4–63.5	79829
*GPR137*	G protein-coupled receptor 137	11	63.8–63.9	56834
*ATG2A*	ATG2 autophagy related 2 homolog A	11	64.4–64.5	23130
*PPP2R5B*	protein phosphatase 2, regulatory subunit B',	11	64.4–64.5	5526
*EHBP1L1*	EH domain binding protein 1-like 1	11	65.1–65.2	254102
*ADRBK1*	adrenergic, beta, receptor kinase 1	11	66.7–66.9	156
*RAD9A*	RAD9 homolog A	11	66.9–67.0	5883
*CABP4*	calcium binding protein 4	11	66.9–67.0	57010
*ACY3*	aspartoacylase 3	11	67.1–67.2	91703
*CTTN*	Cortactin	11	66.9–67.0	2017
*C11orf30*	chromosome 11 open reading frame 30	11	75.8–76.0	56946
*RAB30*	RAB30, member RAS oncogene family	11	82.3–82.5	27314
*TMEM126B*	transmembrane protein 126B	11	85.0–85.1	55863
*PICALM*	phosphatidylinositol binding clathrin assembly protein	11	85.3–85.5	8301
*NEK3*	NIMA (never in mitosis gene a)-related kinase 3	13	51.6–51.7	4752
*UBL3*	ubiquitin-like 3	13	29.2–29.4	5412
*TDRD3*	tudor domain containing 3	13	59.8–60.1	81550
***Downregulated genes within regions of relative copy number decrease***				
*RAD52*	RAD52 homolog	12	0.8–1.0	5893
*FBXL14*	F-box and leucine-rich repeat protein 14	12	1.5–1.6	144699
*CACNA2D4*	calcium channel, voltage-dependent, alpha 2/delta subunit 4	12	1.7–1.9	93589
*ITFG2*	integrin alpha FG-GAP repeat containing 2	12	2.7–2.9	55846
*LRRC23*	leucine rich repeat containing 23	12	6.8–6.9	10233
*H3F3B*	H3 histone, family 3B	17	71.2–71.3	3021
*SOCS3*	suppressor of cytokine signaling 3	17	73.8–73.9	9021
*RNF213*	ring finger protein 213	17	75.9–76.0	57674

### Commonly dysregulated genes in PPP tolerant cell lines

**Figure 4:**
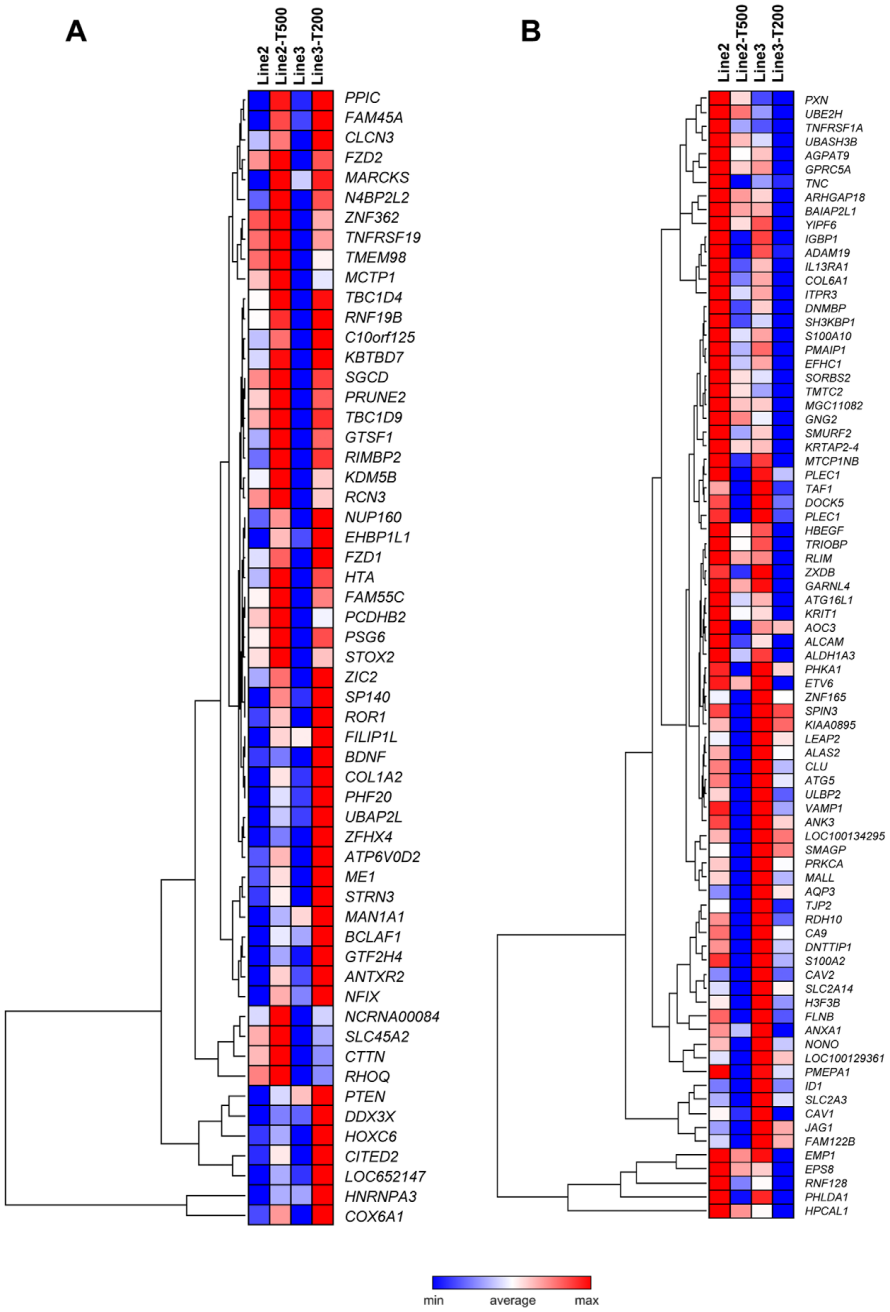
Heat maps and hierarchical clustering of genes in gene list H. The heat maps show (A) upregulated and (B) downregulated genes in PPP tolerant (Line2-T500 and Line3-T200) cells as compared to parental Line2 and Line3 cells. Relative expression levels are indicated according to the colour scale and further gene information is given in Table S5.

Comparison of expression profiles in PPP tolerant and parental Line2 and Line 3 cells revealed many dysregulated genes, in addition to those with concomitant copy number alterations. Two different sets of criteria were applied for selection. Altogether 561 genes were commonly dysregulated in both lines showing at least 2-fold difference in expression level between tolerant and parental cell lines. The 286 up-regulated and 275 down-regulated genes are detailed in Table S4 (Gene list U). Using the more stringent criteria (detailed in material and methods) from 3,440 informative transcripts we identified 78 up-regulated and 115 down-regulated genes (gene list H) that were common to Line2-T500 and Line3-T200 (Figure 4; Table S5).

A functional annotation of gene lists U and H is detailed in Tables S6 and S7. From gene list U up-regulated genes were identified in the categories cell death, down-regulated genes in cell death, positive regulation of cellular process, cell differentiation, regulation of programmed cell death, positive regulation of metabolic process, regulation of cell differentiation, positive regulation of developmental processes and response to wounding (Table S6). Genes within the category of cell death were detected from both list U and H (Table 3). Among these may be noted up-regulation of *Kiaa0367*, *PTEN*, *MAPK*, *BCL2, BCLAF1*, *CASP1* and *TNFRF19*, and down-regulation of *EMP1*, *SOCS3*, *NAIP, STK17A, PRKCA*, *ALDH1A*, *CLU*, *PHLDA1*, *PMAP*, *TNFRSF1A*, *ATG5* and *ANXA1*. Among these, *MAPK, BCL2, SOCS3, ALDH1A* and *ANXA1* were validated as described below.

**Table 3:** Genes dysregulated in PPP tolerant Line2-T500 and Line3-T200 cells in GO category 0008219 cell death (from gene list U).

Gene symbol	Gene name	Location	Line2-T500	Line3-T200
***Up-regulated***				
*VDAC-1*	voltage-dependent anion channel 1	5q31	up	up
*KIaa0367*	KIAA0367	9p21.13	up	up
*CECR2*	cat eye syndrome chromosome region, candidate 2	22q11.2	up	up
*PTEN*	phosphatase and tensin homolog	10q22.3	up	up
*MAPK* *	Mitogen-activated protein kinase 1	22q11.2	up	up
*BCL2* *	B-cell CLL/lymphoma 2	18q21.33	up	up
*BCLAF1*	BCL2-associated transcription factor 1	6q22-23	up	up
*CASP1*	Caspase-1	11q23	up	up
*HRK*	harakiri, BCL2 interacting protein	12q24.22	up	up
*PHF17*	PHD finger protein 17	4q26-q27	up	up
*FKSG2*	Apoptosis inhibitor	8p11.2	up	up
*EIF5A*	Eukaryotic translation initiation factor 5A	17p13-12	up	up
*TNFRSF19*	tumor necrosis factor receptor superfamily, member 19	13q12.11-12.3	up	up
*ADORA1*	Adenosine A1 receptor	1q32.1	up	up
*Lgals1*	Lectin, Galactoside-binding, soluble, 1 (Galectin 1)	22q13.1	up	up
*PSEN2*	Presenilin 2	1q31-42	up	up
*CRYAB*	crystallin, alpha B	11q22.3-23.1	up	up
*CFLAR*	CASH, CASP8 and FADD-like apoptosis regulator	2q33-34	up	up
***Down-regulated***				
*EMP1*	Epithelial membrane protein	12p12.3	down	down
*SOCS3* *	Suppressor of cytokine signaling 3	17q25.3	down	down
*NAIP*	NLR family, apoptosis inhibitory protein	5q13.1	down	down
*ERCC-6*	DNA excision repair protein ERCC-6, CDNA FLJ13497 fis	10q11.23	down	down
*STK17A*	Serine/Threonine kinase 17A (apoptosis-inducing)	7p12-p14	down	down
*PRKCA*	Protein kinase C, alpha	17q22	down	down
*PMAP*	phorbol-12-myristate-13-acetate-induced protein 1	18q21.32	down	down
*BCLAF1*	BCL2-associated transcription factor 1	6q22-23	down	down
*EFHC1*	Hypothetical protien fli10466	6p12.3	down	down
*CLU*	Clusterin	8p21-12	down	down
*ALDH1A3* *	Aldehyde dehydrogenase 1 family, member 3	15q26.3	down	down
*ABPP*	Amyloid beta (A4) protein	21q21.2	down	down
*MCL-1*	myeloid cell leukemia sequence 1 (bcl2-related)	1q21	down	down
*CTNNAL1*	catenin (cadherin-associated protein), alpha-like 1	9q31.2	down	down
*PHLDA1*	Pleckstrin homology-like domain, family A, member 1	17q22-23	down	down
*KRT-20*	Keratin-20	17q21.2	down	down
*PAWR*	PRKC apoptosis WT1 regulator protein, CDNA FLJ14942 fis	12q21	down	down
*ANXA1* *	annexin A1	9p12-q21.2	down	down
*TRIM35*	Tripartite motif-containing protein 35, IMAGE:4052341	8p21.2	down	down
*TNFRSF1A*	tumor necrosis factor receptor superfamily, member 1A	12p13.2	down	down
*SH3KBP1*	SH3-domain kinase binding protein 1	Xp22.1-21	down	down
*ATG5*	ATG5 autophagy related 5 homolog (S. cerevisiae)	6q21	down	down

*indicates genes validated by siRNA.

### Validation of identified genes by siRNA treatment

To confirm the results of the comparative expression analysis and test the results in a wider range of cell lines, siRNA was used to alter the gene expression and the effect of PPP on cell viability was then measured. The tested hypothesis in this experiment was that the upregulated genes in the tolerant cell lines contribute to the PPP resistance and therefore suppressing them would increase sensitivity to PPP. Reciprocally, we designed an experiment in the parental cell lines and other cancer cell lines by inhibiting genes from the downregulated group. If downregulation contributes to PPP resistance, this experiment would validate the gene identification process. We used two different approaches in building the siRNA library. The first was an unbiased approach based on the measured effect on expression, with the top four genes from the up and downregulated lists from Supplemental Table S4, were chosen. The second, biased, approach identified five genes from Table 3 that we believe to have a clear connection with IGF-1R signaling or with drug resistance. Altogether, six genes that were identified as downregulated in PPP tolerant cell lines and seven genes that were identified as upregulated in PPP tolerant cell lines were chosen for this analysis. The six downregulated genes included the top four downregulated identifiable genes *ALDH1A3* (an aldehyde dehydrogenase involved alcohol and lipid metabolism), *ANXA1* (annexin A1, a Ca2+ dependent phospholipase involved in inflammatory modulation), *TLR4* (toll like receptor 4 involved in pathogen recognition in innate immunity) and *SPIN3* (spindlin family member 3), plus *SOCS3* (suppressor of cytokine signaling 3) as it is known to interact with IGF-1R, and *RAB5A* as it is a member of the Ras oncogene family and so both had potential direct connections with IGF-1R downstream signaling. The four most upregulated genes were: *COX6A1* (a cytochrome oxidase involved in the mitochondrial respiratory chain), *LGALS2* (a soluble galactoside binding lectin), *NFIX* (a transcription factor) and *ME1* (malic enzyme 1). In addition, we included in our library *BCL2* as it is involved in negative regulation of apoptosis, *MAPK* (*ERK2*) as it is involved in IGF-1R downstream signaling and *TAP2,* a member of the ATP binding cassette (ABC) transporter family involved in multi-drug resistance.

The genes downregulated in PPP tolerant cell lines were siRNA silenced in non-tolerant cells Line2, Line3, the melanoma cell line BE and breast cancer cell line MCF7, all known to express IGF-1R and to be responsive to PPP [8]. Following transfection with a non-targeting siRNA control, 500 nM PPP treatment for 48 h caused cell viability of the parental cell lines Line2 and Line3 to decrease by 45% and 55%, BE and MCF7 by 34% and 40%, respectively whereas, as expected, a limited decrease of only 10% was observed with Line2-T500 and Line3-T200 (Table 4). The siRNA treatment alone had no significant effect on cell viability. Downregulation of the top two downregulated genes *ALDH1A3* and *ANXA1* caused a significant increase in resistance to PPP in all non-tolerant cell lines including the two parental cell lines. In addition, treatment with siRNA against *TLR4*, *SPIN3*, *SOCS3* and *RAB5A* increased PPP resistance significantly in at least two of the four tested cell lines for each gene. Of these, at least one of the parental cells showed increased resistance with the exception of *SOCS3* for which Line2 and Line3 showed no change however the other cell lines included, BE and MCF7 both showed significant increased tolerance (Table 4).

**Table 4:** Validation of identified genes by siRNA treatment.

*Downregulated genes*																
		Negative	*ALDH1A3*		*ANXA1*		*TLR4*		*SPIN3*		*SOCS3*		*RAB5A*			
Line2	Mean	0.555	0.718	↑*	0.746	↑*	0.628	↑*	0.582	NS	0.549	NS	0.661	↑		
	SEM	0.0015	0.0163		0.0273		0.0080		0.0295		0.0033		0.0449			
Line3	Mean	0.494	0.529	↑*	0.601	↑*	0.596	↑	0.568	↑*	0.508	NS	0.659	↑*		
	SEM	0.0047	0.0111		0.0284		0.0498		0.0087		0.0060		0.0316			
BE	Mean	0.662	0.732	↑*	0.861	↑*	0.701	NS	0.876	↑*	0.698	↑*	0.799	↑*		
	SEM	0.0073	0.0047		0.0143		0.0383		0.0208		0.0058		0.0407			
MCF7	Mean	0.600	0.768	↑*	0.745	↑*	0.696	↑*	0.674	NS	0.712	↑*	0.673	↑		
	SEM	0.0288	0.0209		0.0192		0.0195		0.0297		0.0105		0.0232			

*Upregulated genes*		Negative	*COX6A1*		*LGALS2*		*NFIX*		*ME1*		*BCL2*		*MAPK*		*TAP2*	
Line2-T500	Mean	0,895	0.736	↓*	0.566	↓*	0.723	↓*	0.706	↓*	0.502	↓*	0.740	↓*	0.870	NS
	SEM	0.0412	0.0078		0.0021		0.0164		0.0052		0.0047		0.0072		0.0349	
Line3-T200	Mean	0.901	0.840	↓	0.768	↓*	0.667	↓*	0.537	↓*	0.811	↓*	0.538	↓*	0.967	↑*
	SEM	0.0159	0.0197		0.0341		0.0171		0.0155		0.0195		0.0167		0.0102	
BE	Mean	0.662	0.539	↓*	0.555	↓*	0.485	↓*	0.461	↓*	0.489	↓*	0.426	↓*	0.695	NS
	SEM	0.0073	0.0153		0.0161		0.0070		0.0049		0.0028		0.0120		0.0175	
MCF7	Mean	0.600	0.354	↓*	0.427	↓*	0.537	↓	0.483	↓*	0.347	↓*	0.548	NS	0.928	↑*
	SEM	0.0288	0.0123		0.0567		0.0108		0.0080		0.0175		0.0164		0.0461	

The PPP sensitivity ratio was calculated as fluorescence from wells treated with PPP to those treated without PPP.

Statistical significance was calculated using the T test compared to the wells treated with negative control siRNA.

↓  =  Trend in decreased tolerance to PPP, p<0.1; ↓*  =  Significant decreased tolerance to PPP, p<0.05.

↑  =  Trend in increased tolerance to PPP, p<0.1; ↑*  =  Significant increased tolerance to PPP, p<0.05.

NS  =  No statistically significant change.

The genes upregulated in PPP resistant cell lines were siRNA silenced in the cells Line2-T500 and Line3-T200 to determine if a PPP re-sensitization could be achieved. As an alternative experimental model, the same genes were silenced in the BE and MCF7 cells to see if this could increase sensitivity to PPP in non-resistant cell lines. Treatment of the tolerant cell lines Line2-T500 and Line3-T200 with siRNA against the upregulated genes decreased PPP tolerance except for the striking exception of *TAP2*, the ABC transporter which is part of the multidrug resistant family of membrane channels. For *TAP2*, no significant change was seen with Line2-T500 however an increase in tolerance was observed for Line3-T500 which could be explained with its tolerance to a lower level of PPP. The same pattern was mirrored in the siRNA treatment of BE and MCF7 against the upregulated genes. A significant sensitization to PPP was observed in the majority of cases again, except for *TAP2* where MCF7 showed increased tolerance to PPP and BE showed no significant change (Table 4). Taken together, the results from this experiment demonstrated a clear decrease in PPP sensitivity after silencing the downregulated genes and a clear increase in PPP sensitivity after silencing of the upregulated genes.

**Figure 5:**
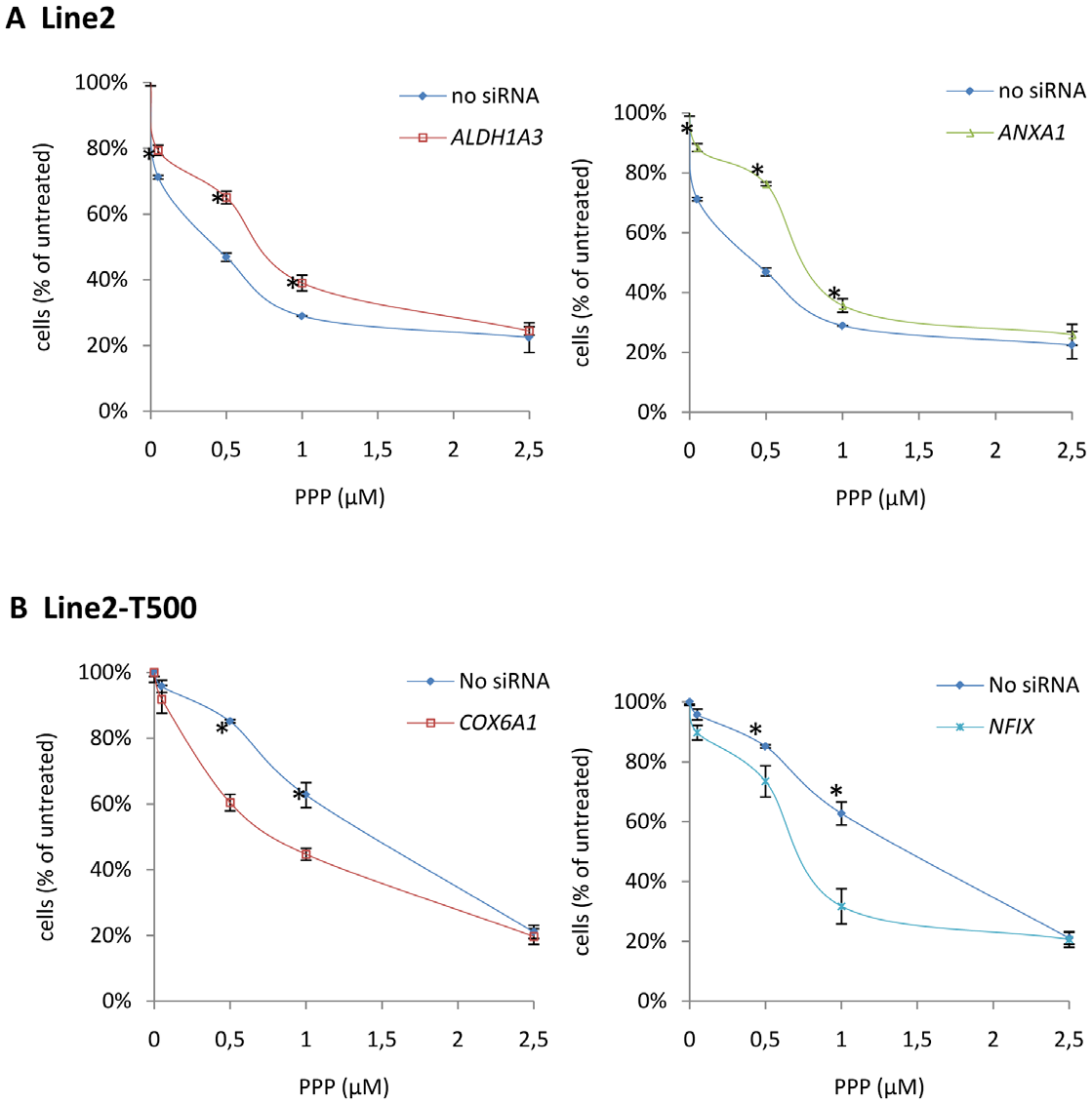
PPP sensitivity curves following siRNA treatment. (**A**) Line2 cells were treated with siRNA for the downregulated genes *ALDH1A3* or *ANXA1*, and (**B**) PPP tolerant Line2-T500 cells were treated with siRNA for the upregulated genes *COX6A1* or *NFIX*. Cell lines transfected with siRNA were incubated with 0, 0.05, 0.5, 1 and 2.5 µM PPP for 48 hours and cell viability assessed using alamarBlue as described in the materials and methods. Cell numbers were interpolated from a standard curve and plotted relative to untreated cells. Statistical significance, p≤0.05, as calculated using a two tailed T test is indicated by *.

To produce a more complete picture of the effect of these genes on sensitivity to PPP, siRNA transfected Line2 and Line2-T500 cells were treated in a PPP dose response experiment for 48 hours. As shown in Figure 5 treatment of Line2 with siRNA for two genes detected as downregulated in resistant cell lines, *ALDH1A3* and *ANXA1*, demonstrated increased resistance following downregulation and incubation with PPP concentrations up to 1 µM. Significantly increased resistance was detected at 0.05, 0.5 and 1 µM PPP treatment but not at 2.5 µM PPP for either gene. This confirms the initial siRNA studies and demonstrates a role in resistance of PPP at a range of concentrations. As shown in Figure 5, treatment of Line2-T500 with siRNA for two genes detected as upregulated in resistance cell lines, *COX6A1* and *NFIX*, demonstrated decreased resistance following downregulation and incubation with PPP at concentrations up to 1 µM, with no difference seen at 2.5 µM. These differences were significant for both genes at 0.5 and 1 µM PPP and again confirm and expand on the initial siRNA studies.

To confirm that the siRNA was decreasing expression of the relevant genes in the control and resistant cell lines, Line2 and Line2-T500 were transfected on a larger scale, the RNA extracted and reverse transcribed into cDNA. This enabled qRT-PCR measurement of the mRNA level to compare untreated with siRNA treated and to confirm that the siRNA is functional. qRT-PCR products were separated by agarose gel electrophoresis to confirm that they migrated at the expected size and melting curve analysis confirmed that all amplification products from a primer pair had the same melting point. Of the downregulated genes, 4 out of 6 showed decreased mRNA expression with siRNA treatment; *ALDH1A3*, *ANXA1*, *TLR4* and *RAB5A* (Table S1). Of the remaining two, *SPIN3* and *SOCS3*, no conclusive results were achieved, despite adequate amplification of *GAPDH* in these samples, suggesting a problem with primer design. Of the upregulated genes, 5 out of 7 showed siRNA treatment decreased mRNA levels; *COX6A1*, *NFIX*, *ME1*, *MAPK* and *TAP2* (Table S1). No conclusive results were achieved with the remaining two genes, *LGALS2* and *BCL2*, due to primer design and inadequate amount of sample, respectively. This demonstrates that the siRNA was specifically decreasing expression of the relevant genes in all cases where the PCR assay worked.

### Global hypomethylation in PPP tolerant cell lines

**Figure 6:**
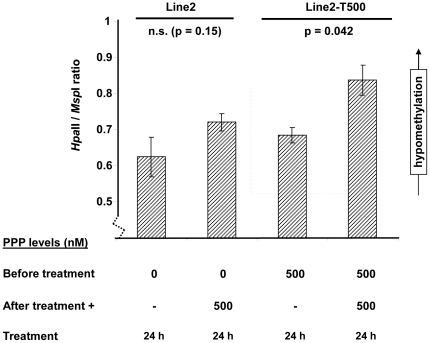
Global hypomethylation after PPP treatment of Line2 cells. Methylation at CCGG sites measured by LUMA in PPP sensitive Line2 and tolerantLine2-T500 cell**s**. The Y-axis shows the *Hpa*II/*Msp*I ratio, which correlates inversely to DNA methylation levels. Line2 cells cultured in PPP free media were short-term treated for 24 h with or without 500 nM PPP. Line2-T500 cells cultured in 500 nM PPP were similarly treated for 24 h by adding 500 nM PPP (total concentration 1000 nM). Peak heights were plotted with error bars ± SD. P-values were calculated using the t-test, n.s.  =  not statistically significant.

Genome-wide methylation at CCGG sites were investigated by LUMA in parental Line2, Line3 and their derivatives Line2-T500 and Line3T200. We observed significant hypomethylation in Line2-T500 following PPP treatment (p = 0.04, Figure 6). In Line2 cells, a similar trend of hypomethylation was observed after treatment, however the difference did not reach statistical significance. This trend of hypomethylation was reversed in Line2-T500 (without adding treatment) but retained in Line2-T500 after treatment with 500 nM PPP. There was no difference in DNA methylation between parental Line3 and tolerant lines (data are not shown). The dose of PPP used in the methylation analysis was based on our previous results which showed that parental cell Line2 and Line3 responded to PPP treatment with IC_50_ values of around 0.05 and 0.1 µM, respectively [7], and similar toxicity of PPP for parental cells was also observed in the current study (Figure 6). As treatment of parental cells with high dose of 1000 nM PPP was not possible, it cannot be determined whether the observed hypomethylation was related to the acquired tolerance or if it was merely a result of the high PPP dose during treatment.

## Discussion

Drug resistance may occur as an acquired phenotype after long-term treatment. It may also be characteristic of the primary tumor, either as a feature at initial diagnosis or following selection of tolerant tumor cells. Given the techniques applied, the DNA copy number abnormalities observed in our study are expected to be representative for the majority of cells in each line studied. In this study we focus our attention mainly on molecular changes related to PPP tolerance. However, some striking aberrations common to parental and tolerant cells deserve some discussion as they may have a permissive role in resistance development. The oncogenes *MYC* and *PVT1* in 8q24.1 were amplified or gained in all lines (Figure 1). PVT1 is a c-Myc activator implicated in murine tumors and in the same pathways as c-Myc [32] and c-Myc is a transcriptional regulator affecting cellular processes such as growth, metabolism, division and apoptosis. In animal models, lack of IGF-1R did not prevent tumor formation or progression when *c-myc* was induced [33] and ectopic expression of c-Myc could override the suppressive effects of p16, p21 and p27 to promote cell cycle progression [34]. In this context, the homozygous loss in 9p21.3 observed in Line2/Line2-T500 cells is also noteworthy as it includes *CDKN2A* encoding the cell cycle regulator p16 and p14ARF. Gil *et al*. demonstrated that c-Myc can promote cell cycle progression regardless of the presence of growth factors [35]. In addition, it has been shown that *CDKN2A* loss can contribute towards resistance to targeted therapy in acute lymphoblastic leukemia induced by *BCR-ABL* translocation [36].

It is known that PPP can be non-enzymatically converted to the microtubule inhibitor podophyllotoxin (PPT) [37] with a PPP:PPT steady state equilibrium of 97.5:2.5%. This means that up to 2.5% of PPP converts to PPT. Consistently we have detected small amounts of PPT in culture media incubated with PPP but have not observed any detectable levels of PPT in blood samples from animals (unpublished data) treated with high doses of PPP [10]. In agreement with possible PPT exposure of PPP treated cells (≤12.5 nM in Line2-T500; ≤5 nM in Line 3-T200) we observed commonly altered expression of tubulin or microtubule associated genes in Line2-T500 and Line3-T200 cells. These included over-expression of *MARK1* (9.3 fold change) and *TUBA4A* (4.4 fold) and under-expression of *EML5* (0.19 fold) and *MICAL2* (0.26 fold).

In this study we combined two separate analysis methods array-CGH which detects DNA copy number alterations with expression array which provides information at the transcriptional level. We identified genes that were altered in both studies and which are therefore strong candidates for contributing to PPP tolerance. Amplification and gain involving 5q11.2, gain of 11p12 and 11q12.1-q14.3 and gain of 13q33.3-qter were found as commonly acquired aberrations in Line2-T500 and Line3-T200. Among these the narrow amplification of 5q11.2 in Line3-T200 is the most prominent alteration, but was not paralleled with increased expression for informative genes in the amplified region. However, up-regulation was observed for genes in the gained 11p12 region including *ACP2*, *CUGBP1* and *FNBP4* and in the gained 11q12.1-q14.3 interval for *ATG2A*, *CABP3*, *NAALAD2, TncRNA*, *CTTN*, *RAB30* and *EHBP1*. In addition, under-expression of genes from the distal lost 11q region was observed for *UBASH3B, ZC3H12C*, *RICS* and *ETS1*. The gain of 13q33.3-qter was paralleled by up-regulation of *TMCO3* and *KDELC1.*

Considering expression profiles, the most pronounced alteration identified was the cytochrome c oxidase subunit 6A1 (*COX 6A1*) on 12q. COX 6A1 is a suppressor of apoptosis by inhibiting *BAX*-induced apoptosis [38]. The increase in *COX6A1* expression was confirmed to contribute to PPP resistance after suppression of expression by siRNA in the tolerant cell lines and it was demonstrated that this effect was not limited to the paired tolerant cell lines. Furthermore, genes within the functional category cell death were over-represented among both up and down regulated genes.

IGF-1R is a cell surface receptor tyrosine kinase (RTK) that is substantially expressed in malignant tissues and plays a crucial role in growth and survival of cancer cells but is not absolutely required for normal cell growth [39]. Targeting the IGF-1R is today an attractive concept in oncology and many pharmaceutical companies are developing anti-IGF-1R agents, of which several are now in clinical trials. A substantial part of the anti-neoplastic effects of PPP have been proposed to occur through the IGF-1R. PPP was not only reported to attenuate IGF-1R activity but also to downregulate the receptor [17]. This effect may be important for its strong anti-tumor efficacy and is consistent with the current concept that inhibition of IGF-1R phosphorylation only decreases proliferation of tumor cells whereas in order to promote massive apoptosis and tumor regression the receptor needs to be downregulated.

Insensitivity to drug-induced apoptosis plays an important role in acquired anticancer drug resistance. Different strategies applied to inhibit IGF-1R expression or function have resulted in blocking tumor growth and metastasis and have enhanced sensitivity to cytostatic drugs and irradiation [39], [40]-[41]. Therefore one could expect that in the case of PPP, an acquired resistance mechanism would only barely evolve. The balance between the pro-survival and pro-apoptotic factors encoded by the genetic alterations associated with the tolerant phenotype in the Line2-T500 and Line3-T200 must have allowed the establishment of limited resistance to PPP over the 80 week period.

We found significant hypomethylation after PPP treatment in Line2-T500 cells and there was also a trend of hypomethylation in Line2 cells. Based on experiments performed it could not be concluded whether the hypomethylation was related to the acquired PPP tolerance or if they resulted from the high dose of PPP. Hypermethylation of CpG islands at tumor suppressor genes switches off these genes whereas global hypomethylation leads to genomic instability and activation of oncogenes. Considering the limited changes in the gene copy number in tolerant versus parental cell lines it is possible that epigenetic activation of oncogenes is able to substitute for the IGF-1R signaling to contribute to the development of PPP tolerance. This factor, especially in relation to the inhibitory effect of PPP on IGF-1R should be investigated in more detail.

DNA copy number imbalances between parental and PPP treated cells were limited suggesting that PPP treatment does not induce high level amplification of drug resistance genes. Abnormalities related to PPP tolerance observed in chromosomal regions 3q26, 10p14, 11p11, 11q11, 12p13, 13q12, 13q13 and 17q24 correlated with altered gene expression. This correlation increases the probability that genes in these regions were subjected to selective pressure to contribute to drug tolerance. An additional set of commonly dysregulated genes were identified without concomitant alteration in DNA copy number. Genes functionally related to cell death were significantly dysregulated.

The mechanism behind acquired PPP resistance is clearly very complex, with a large number of dysregulated genes. For this reason it is impossible to assess the contribution of each gene to the resistant phenotype. Nevertheless, by using a small siRNA library we have validated the process used to identify dysregulated genes. The siRNA experiments clearly demonstrated that suppression of expression of the top identifiable down and upregulated genes had clear effects on sensitivity to PPP matching their alteration in expression in the tolerant cell lines. These effects were not limited to the cell lines in the study but extended to the two other cancer cell lines tested, BE and MCF7. The four additional genes tested that were chosen from the up and downregulated lists due to connections with IGF-1R function (*SOCS3*, *RAB5A BCL2* and *MAPK*) also showed effects appropriate to their alteration in the tolerant cell lines.

In summary, in the present study we used an *in vitro* approach to identify the genetic changes induced by prolonged exposure to an IGF-1R inhibitor and the possible mechanisms of acquired resistance to such treatment. By analysing the DNA copy number alterations in combination with transcripts expression array we could identify the genes that are strong candidates for contributing to PPP tolerance. Moreover, we could validate the identification process by silencing them both in the resistant cell lines model and in naive cell lines.

## Supporting Information

Figure S1 Representative SKY karyotypes of parental Line2 and Line3 cells. Each chromosome is shown in SKY painting colors (left) and in SKY classification pseudo-colors (right). Chromosome numbers are indicated below, as well as to the right of derivatives composed of two or more different chromosomes.
(2.44 MB TIF)

Figure S2 Schematic illustrations of copy number alterations detected by metaphase CGH in parental and tolerant cells. Copy number alterations detected by metaphase CGH in parental Line2 and Line3 cells (C) as compared to PPP tolerant derivatives at different levels of PPP in Line2 (40, 100, 300, 400 and 500 nM PPP) and Line3 (40 and 100 and 200 nM PPP at two different time-points) cells. Alterations are indicated along the chromosome ideograms using bars to the left for losses, to the right for gains, and in bold for amplifications.
(0.29 MB TIF)

Table S1 Results and primer details for qRT-PCR analyses of siRNA.
(0.02 MB PDF)

Table S2 Copy number abnormalities by array-CGH in parental and PPP tolerant Line2, Line3 cells.
(0.02 MB PDF)

Table S3 Homozygous losses and narrow narrow amplifications detected.
(0.02 MB PDF)

Table S4 Gene list U, commonly up-regulated or down-regulated genes in Line2-T500 and Line3-T200 cells (>2-fold difference between parental and PPP tolerant cells).
(0.15 MB PDF)

Table S5 Gene list H, up and down-regulated genes in PPP tolerant Line2-T500 and line3-T200 cells (including genes with >2-fold difference vs parental cells).
(0.03 MB PDF)

Table S6 Ontological categories generated from common up-regulated and down-regulated genes in PPP tolerant Line2-T500 and Line3-T200 cells (by DAVID, from gene list U).
(0.04 MB PDF)

Table S7 Ontological categories generated from common up- and down-regulated genes in PPP tolerant Line2-T500 and Line3-T200 cells (by DAVID, from gene list H).
(0.01 MB PDF)
