# Supplementary figures and images for: Molecular Characterization of Acquired Tolerance of Tumor Cells to Picropodophyllin (PPP)
Source: PLoS One. 2011 Mar 14;6(3):e14757. doi: 10.1371/journal.pone.0014757 (PMC3056661; doi:10.1371/journal.pone.0014757)

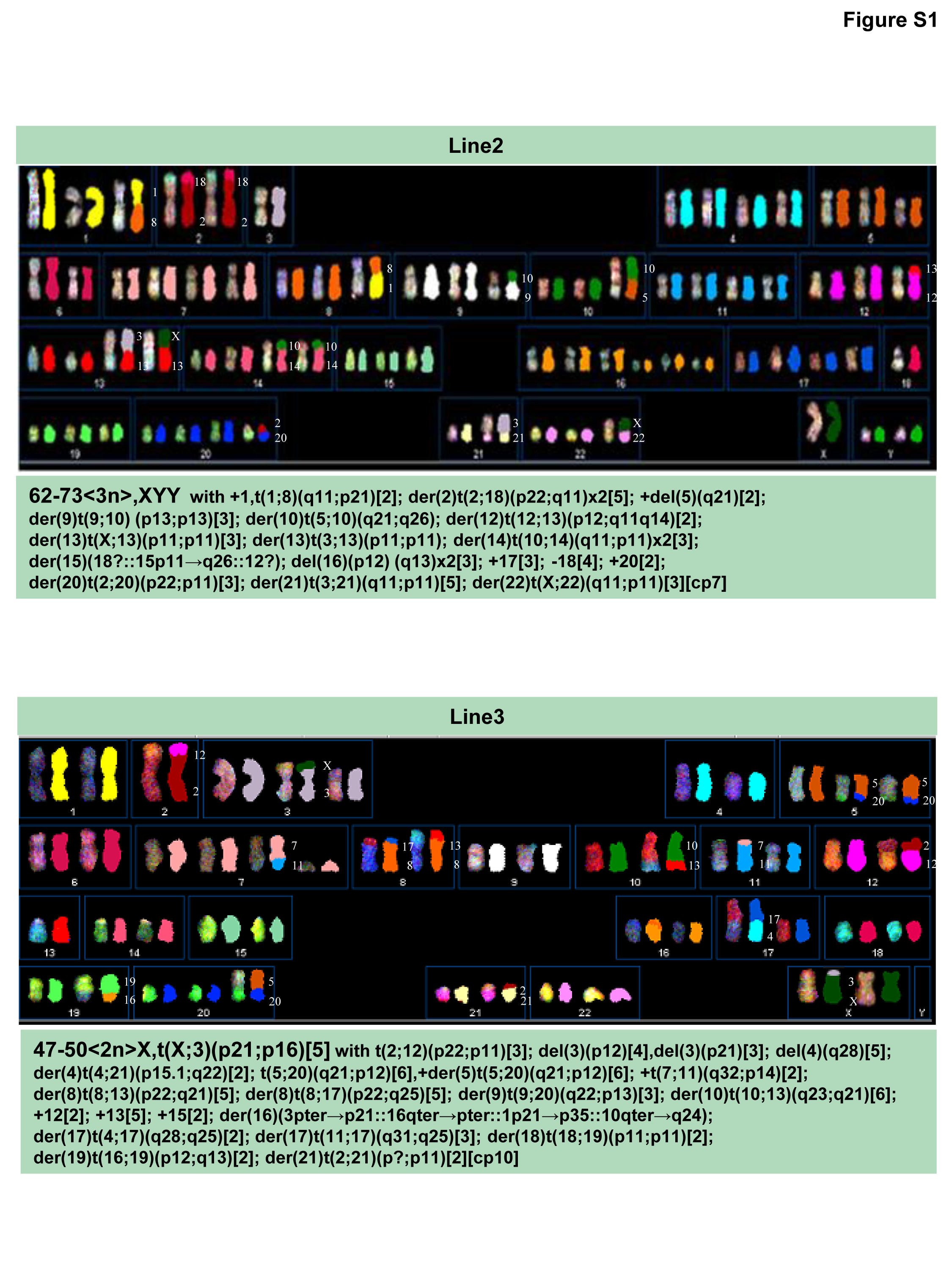

Supplement: Figure S1 — Representative SKY karyotypes of parental Line2 and Line3 cells. Each chromosome is shown in SKY painting colors (left) and in SKY classification pseudo-colors (right). Chromosome numbers are indicated below, as well as to the right of derivatives composed of two or more different chromosomes. (2.44 MB TIF) [file pone.0014757.s001.tif]

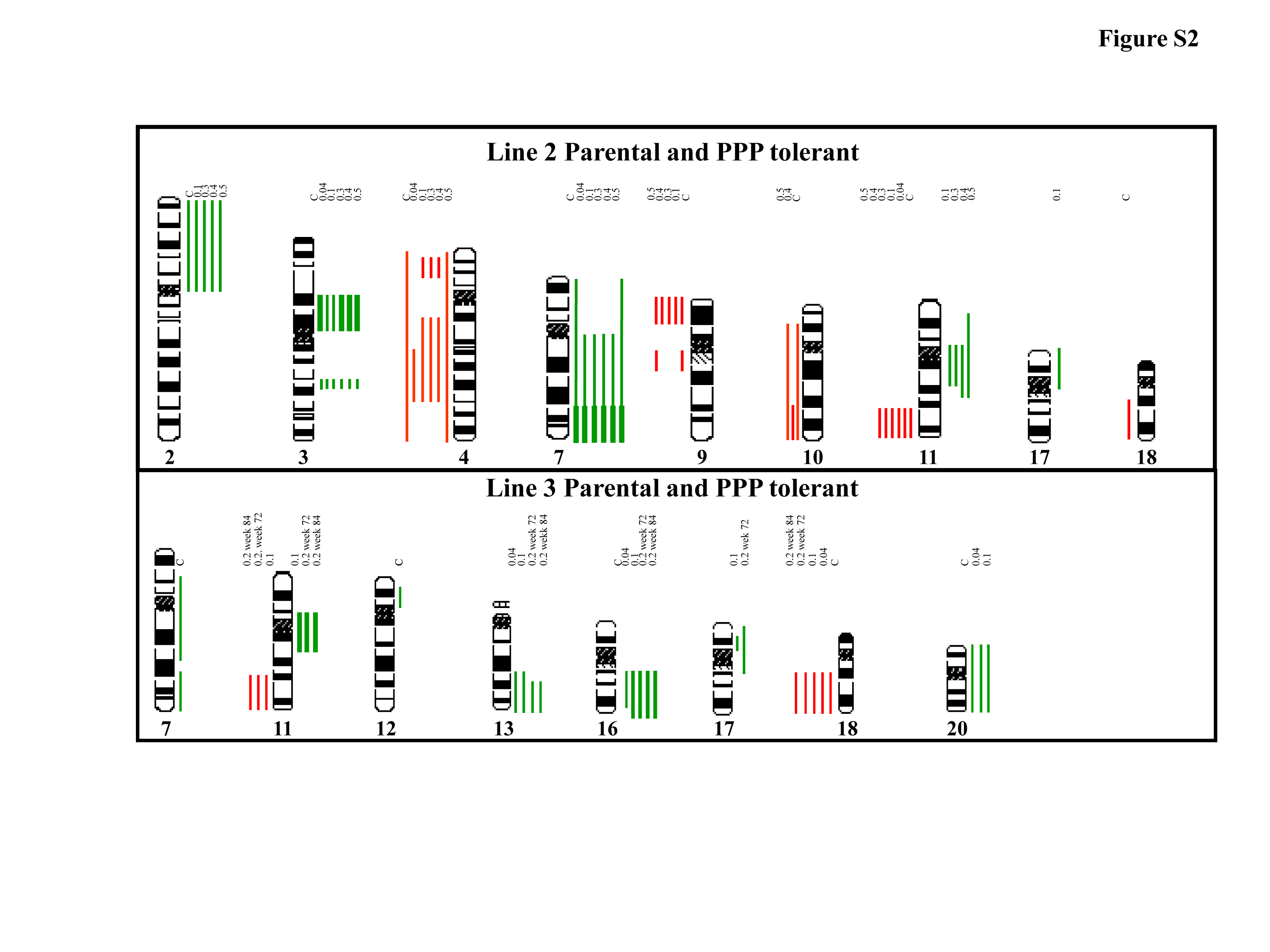

Supplement: Figure S2 — Schematic illustrations of copy number alterations detected by metaphase CGH in parental and tolerant cells. Copy number alterations detected by metaphase CGH in parental Line2 and Line3 cells (C) as compared to PPP tolerant derivatives at different levels of PPP in Line2 (40, 100, 300, 400 and 500 nM PPP) and Line3 (40 and 100 and 200 nM PPP at two different time-points) cells. Alterations are indicated along the chromosome ideograms using bars to the left for losses, to the right for gains, and in bold for amplifications. (0.29 MB TIF) [file pone.0014757.s002.tif]
