# Supplementary material for: Molecular Characterization of Acquired Tolerance of Tumor Cells to Picropodophyllin (PPP)
Source: PLoS One. 2011 Mar 14;6(3):e14757. doi: 10.1371/journal.pone.0014757 (PMC3056661; doi:10.1371/journal.pone.0014757)
Supplement: Table S1 — Results and primer details for qRT-PCR analyses of siRNA. (0.02 MB PDF) [file pone.0014757.s003.pdf]

Table S1. Results and primer details for qRT-PCR analyses of siRNA.

Gene		Primer sequences 5'-3'	Product size	Melting point	Effect of siRNA
Downregulated genes					
ALDH1A3	F	GCGAATAGCACCGACTATGG	136 bp	81 °C	37.8%
	R	ATGGAGCCTGTGCATAGAGG			
ANXA1	F	GACCGATCTGAGGACTTTGG	129 bp	79 °C	21.1%
	R	GCTTCTTGGTGGTAACCATGG			
TLR4	F	AATCCCCTGAGGCATTTAGG	100 bp	77 °C	63.6%
	R	CCCCATCTTCAATTGTCTGG			
SPIN3	F	GATGGCTCCAAGAGAACTGG	142 bp	75 °C	n/a
	R	TTGGCAAATTTCAAGATGACC			
SOCS3	F	CTACTGGAGCGCAGTGACC	112 bp	n/a	n/a
	R	GCTGAGCGTGAAGAAGTGG			
RAB5A	F	ATTTCCAGGAAGCACAGTCC	144 bp	74 °C	29.4%
	R	TTGCTCCTGGATTTTGTGG			
Upregulated genes					
COX6A1	F	ACGGAGAGCACGAGAGACC	122 bp	81.5 °C	4.3%
	R	TGGAAGTGGATTCACATGAGG			
LGALS2	F	AAGATCACAGGCAGCATCG	117 bp	81 °C	n/a
	R	GCAGACAATGGTGGATTCG			
NFIX	F	GAGAGCCCTGTTGATGACG	140 bp	79 °C	9.0%
	R	CTGCAGAAGTCCAGCTTTCC			
ME1	F	CTATGTGTTCCCTGGAGTTGC	137 bp	86 °C	2.0%
	R	GATAAAGCCGACCCTCTTCC			
BCL2	F	GCCTTATGCATTTGTTTTGG	145 bp	74 °C	n/a
	R	TTCGGATCTTTATTTCATGAGG			
MAPK	F	CACCAACCTCTCGTACATCG	133 bp	81 °C	56.5%
	R	CAGGGTTCTCTGGCAGTAGG			
TAP2	F	CGCCTTCTTCTTCCTTGTCC	101 bp	78 °C	47.2%
	R	CATGGGGGTCAAAATCACC			
Reference gene					
GAPDH	F	GGGAAGCTTGTCATCAATGG	126 bp	82 °C	n/a
	R	CTCCATGGTGGTGAAGACG			

F = Forward; R = Reverse; n/a = not available or not applicable
