# Supplementary material for: Molecular Characterization of Acquired Tolerance of Tumor Cells to Picropodophyllin (PPP)
Source: PLoS One. 2011 Mar 14;6(3):e14757. doi: 10.1371/journal.pone.0014757 (PMC3056661; doi:10.1371/journal.pone.0014757)
Supplement: Table S2 — Copy number abnormalities by array-CGH in parental and PPP tolerant Line2, Line3 cells. (0.02 MB PDF) [file pone.0014757.s004.pdf]

Table S2. Copy number abnormalities by array-CGH in parental and PPP tolerant Line2 and Line3 cell lines.

	Losses		Gains	
	Parental	PPP tolerant	Parental	PPP tolerant
Line2 and Line2-T500				
Chr 1	1p21.1-p22.1	1p21.1-p22.1	1p32.3-p35.1; p13.3-q21.1	1p32.3-p35.2; p11.2-p13.3
Chr 2	-	-	2p11.2-pter; q14.3	2p12-pter
Chr 3	3q23-q26.31	-	3p11.1-p14.1/**p12.2-p12.3, p13-p14.1;** q21.2-q22.3/**q22.3**	3p11.1-p14.2; q21.2-q22.3
Chr 4	4p11-q15.32; q13.2-qter	4p11-p15.32; q13.1-q31.3	4q12	-
Chr 5	5q12.1	5p14.3-pter	-	-
Chr 6	-	6p21.31-qter	6p24.3-pter	6p24.3-pter
Chr 7	-	-	7pter-q11.21; q11.22-q31.1; q31.33-qter/**q32.1-q36.1**	7q32.1-q34; q35-qter/**q33**
Chr 8	-	-	**8q21.2**; q24.13-qter	**8q21.2;** q24.13-qter
Chr 9	9p13.2; p21.1-pter ***/p21.1-p21.3 hz***	9p13.2; p21.1-pter /***p21.1-p21.3 hz*** ; p11.3-q12	9q34.11-qter	-
Chr 10	10p12.32-q21.3; q22.1-q22.2;q23.2--q23.33/***q23.33-q24.1 hz*** ; q24.33-qter	10q23.33-q24.2/***q23.33-24.2 hz*** ; q24.32-q26.3	-	10p12.32-pter; q22.2-q23.2
Chr 11	11q22.1-qter	11q22.1-qter	11p15.5-pter	-
Chr 12	12p12.3-p13.31	***12p12.3-p13.2 hz*** ; p13.31-pter	12p11.1-p12.1; q23.3-qter	12p11.1-p12.3; q23.3-q24.31
Chr 13	13q31.1	-	-	13q31.3-qter
Chr 14	-	-	-	-
Chr 15	-	-	-	-
Chr 16	16q22.3-q24.1	16q22.3-q23.1; q23.1-qter	16p11.2-q21; q22.1-q22.3	16p11.2-q22.3
Chr 17	-	-	17q22-q23.2	17q22-q23.2
Chr 18	18q12.3-q21.2; q21.31-q22.3	18q12.3-q21.2; q21.31-q22.3	18p11.21-pter; q11.1-q11.2	18q11.1-q11.2; q21.2-q21.31; q22.3-qter
Chr 19	19p13.2; q13.42-qter	19p13.2; q13.42-qter	-	-
Chr 20	***20p12.1 hz***	***20p12.1 hz***	20q11.1-q13.2; q13.31-qter	20q11.1-qter
Chr 21	-	21q11.2-q22.3	21q22.3-qter	-
Chr 22	-	-	22q11.1-q12.3; q12.3-q13.2	-
Line3 and Line3-T200				
Chr 1	-	-	1q41-q43	1q21.1-q25.3; q32.1-q32.3; q41-qter
Chr 2	2p25.1; p13.1	2p25.1; p13.1	-	-
Chr 3	3p14.2	3p12.3-p13	-	3q27.3-qter
Chr 4	4q22.1; q22.3-q31.21	4q22.1	**-**	4p15.2-pter
Chr 5	-	-	-	5p15.1-pter; p13.3-q11.2/**q11.2**

Chr 6	6q26-qter	6q26-qter	-	-
Chr 7	7q31.1	7q31.1; q35	7p11.22-p13-/**p12.3-p13**; **q21.13**; q32.3-qter	**7p12.3-p13**; q21.13
Chr 8	-	-	**8q24.21**	**8q24.21;** q24.22; q24.3
Chr 9	-	-	9q34.3	-
Chr 10	-	-	10q11.2-q23.31	10q11.2-q11.23; q22.1-q23.31
Chr 11	-	11q21-q22.1; q22.1-qter	-	11p11.12-p12; q12.1-q21
Chr 12	-	-	12p11.1-pter/**p11.23-p12.1**; q12-q13.3	12p11.1-p12.1/**p12.1**; q12-q13.3
Chr 13	13q11.1-q14.2	13q11.1-q11.2	13q21.31-q31.1; q31.3-qter	13q21.31-qter
Chr 14	-	-	14q11.2	-
Chr 15	-	-	15q25.3	-
Chr 16	***16p13.2 hz*** ; q23.3	***16p13.2 hz*** ; q23.3-qter	16p11.1-q23.3/**q14-q23.3**	16q11.2-q14; **q14-q23.3**
Chr 17	-	17q23.2	17p13.1-pter; q23.2-q25.3	17q11.2; q12
Chr 18	18q11.2-qter/***q14 hz***	18q11.2-qter	-	18q12.1
Chr 19	-	-	-	-
Chr 20	20p12.1	20p12.1	20p12.1-pter; q11.23-qter	20p12.1-pter; q11.23-qter
Chr 21	-	-	21q22.11	-
Chr 22	22q12.2	22q12.2	-	22q12.3; q13.32-q13.33

Homozygous loss (hz) is indicated in italic and bold; Amplifications are marked in bold
