# Supplementary material for: Molecular Characterization of Acquired Tolerance of Tumor Cells to Picropodophyllin (PPP)
Source: PLoS One. 2011 Mar 14;6(3):e14757. doi: 10.1371/journal.pone.0014757 (PMC3056661; doi:10.1371/journal.pone.0014757)
Supplement: Table S3 — Homozygous losses and narrow narrow amplifications detected. (0.02 MB PDF) [file pone.0014757.s005.pdf]

Table S3. Homozygous losses and narrow amplifications detected.

Cytogenetic interval of alterations	Extent of alteration: starting clone	starting position	ending position	Size Mb	Max log2 ratio in Line2: Parental	T500	Max log2 ratio in Line 3: Parental	T200
Narrow amplifications								
3p12.2-p12.3	RP11-465P18	78566157	81901570	3.3	**1.01**	n.r.	n.r.	n.r.
3p13-p14.1	RP11-582M4	69089544	73418171	4.3	**1.01**	n.r.	n.r.	n.r.
5q11.2	RP11-164L2	53790179	58456855	4.7	n.r.	n.r.	n.r.	**1.97**
7p12.3-p13	RP11-804G9	45101449	48191709	3.1	n.r.	n.r.	**1.61**	**1.51**
7q21.13	RP11-462N5	87558480	87731845	0.17	**1.20**	n.r.	n.r.	n.r.
7q32.1-q34	RP11-329O5	126388390	141547420	15.2	**1.51**	n.r.	n.r.	n.r.
7q33	*RP11-237E23*	*132901984*	*133493650*	*0.6*	***1.37***	***1.11***	n.r.	n.r.
7q36.1-q36.3	RP11-765I1	147532537	158624831	11.1	**1.49**	n.r.	n.r.	n.r.
8q21.2	RP13-580M19	86638084	86898973	0.3	**1.50**	**1.14**	n.r.	n.r.
8q24.21	RP11-367L7	128528775	129838020	1.3	n.r.	n.r.	**2.84**	**2.39**
12p11.23-p12.1	RP11-729I10	21773995	27395869	5.6	n.r.	n.r.	**1.16**	n.r.
12p12.1	*RP11-679N16*	*25756675*	*26380314*	*0.6*	n.r.	n.r.	***1.36***	***1.01***
16q21-q22.1	RP11-467L24	62921783	69356635	6.4	n.r.	n.r.	**1.41**	**1.37**
16q22.3	RP11-681H8	72300040	76987533	4.7	n.r.	n.r.	**1.17**	**1.10**
16q23.1-3	RP11-556H2	77608417	81176355	3.6	n.r.	n.r.	**1.13**	**1.26**
Homozygous losses								
9p21.3	RP11-354P17	21647433	23945955	2.3	**-2.12**	**-2.21**	n.r.	n.r.
9p21.2	RP11-536F2	25553811	27959284	2.4	**-1.97**	**-1.94**	n.r.	n.r.
9p21.1	RP11-264J11	28670844	28849845	0.2	**-1.20**	**-1.58**	n.r.	n.r.
10q23.33-q24.1,(	RP11-706F14	96513911	97386402	0.9	**-2.69**	**-1.49**	n.r.	n.r.
12p13.1-p13.2	RP11-604C17	10134880	13489626	3.3	n.r.	**-1.00**	n.r.	n.r.
12p13.2	*RP11-15O2*	*10568361*	*10899984*	*0.3*	***-1.22***	***-1.11***	n.r.	n.r.
12p12.3	RP11-109B21	15935526	16531364	0.6	n.r.	**-1.05**	n.r.	n.r.
16p13.2	RP11-524I17	6536174	7019966	0.5	n.r.	n.r.	**-1.32**	**-1.15**
18q21.1	RP11-775E21	44959483	45403379	0.4	n.r.	n.r.	**-2.90**	n.r.
20p12.1	RP11-224A21	14802718	15043738	0.3	**-1.03**	**-1.06**	n.r.	**-1.04**

n.r. = no recorded alterations ouside the range -1 to 1
