# Supplementary material for: Molecular Characterization of Acquired Tolerance of Tumor Cells to Picropodophyllin (PPP)
Source: PLoS One. 2011 Mar 14;6(3):e14757. doi: 10.1371/journal.pone.0014757 (PMC3056661; doi:10.1371/journal.pone.0014757)
Supplement: Table S4 — Gene list U, commonly up-regulated or down-regulated genes in Line2-T500 and Line3-T200 cells (>2-fold difference between parental and PPP tolerant cells). (0.15 MB PDF) [file pone.0014757.s006.pdf]

Table S4. Gene list U, commonly up-regulated or down-regulated genes in Line2-T500 and Line3-T200 cells (>2-fold difference between parental and PPP tolerant cells).

Affymetrix Probe Set ID	Gene Symbol	Gene Title	Chromosome location	Fold change in PPP tolerant cells Line2-T500	Line3-T200	Both
Upregulated genes						
224344_at	*COX6A1**	cytochrome c oxidase subunit VIa polypeptide 1	12q24.2	156.3	8440.9	1148.5
216405_at	---'*	HL14 gene encoding beta-galactoside-binding lectin	22q13.1	163.5	2545.5	645.2
231628_s_at	---	---	---	252.7	577.6	382,0
217676_at	---	Transcribed locus, moderately similar to XP_998898.1	---	47.2	316.5	122.3
231655_x_at	---	---	---	22.7	220.8	70.8
1556824_at	---	Full length insert cDNA clone ZM10C10	---	13.1	334.1	66.0
241575_at	---	---	---	43.3	92.5	63.3
209807_s_at	*NFIX **	nuclear factor I/X (CCAAT-binding transcription factor)	19p13.3	35.4	55.8	44.5
211204_at	*ME1**	malic enzyme 1, NADP(+)-dependent, cytosolic	6q12	8.4	207.2	41.7
1570107_at	---	---	---	18.9	63.8	34.7
1565875_at	---	Full length insert cDNA clone YP86C01	6p22.3	20.3	56.1	33.7
227282_at	*PCDH19*	protocadherin 19	Xq13.3	54.2	17.5	30.8
209357_at	*CITED2*	Cbp/p300-interacting transactivator	6q23.3	8,0	89.4	26.7
1563833_at	---	---	---	14.1	49.2	26.3
234802_at	---	---	---	5.9	100.9	24.3
211161_s_at	*COL3A1*	collagen, type III, alpha 1	2q31	10.3	53.4	23.4
231594_at	---	---	---	7.8	68.7	23.1
217114_at	*LOC652147*	similar to U5 snRNP-specific protein, 200 kDa	---	5.2	82.7	20.7
216500_at	---	HL14 gene encoding beta-galactoside-binding lectin	---	12.0	34.3	20.3
241781_at	*C9orf41*	chromosome 9 open reading frame 41	9q21.13	11.8	32.3	19.6
225524_at	*ANTXR2*	anthrax toxin receptor 2	4q21.21	11.2	31.6	18.8
215505_s_at	*STRN3*	striatin, calmodulin binding protein 3	14q13-21	5.6	60.3	18.5
1554201_at	*CABP4*	calcium binding protein 4	11q13.1	5.4	47.0	15.9
1570382_at	---	---	---	7.7	28.9	15,0
204135_at	*FILIP1L*	filamin A interacting protein 1-like	3q12.1	9.7	19.7	13.8
201668_x_at	*MARCKS*	myristoylated alanine-rich protein kinase C substrate	6q22.2	27.4	7.0	13.8
241771_at	*RIMBP2*	RIMS binding protein 2	---	10.9	15.1	12.8
231040_at	---	CDNA FLJ43172 fis, clone FCBBF3007242	9q22	2.2	68.1	12.3
1554506_x_at	*NAALAD2*	N-acetylated alpha-linked acidic dipeptidase 2	11q14.3-21	43.1	3.4	12.1
1554302_s_at	*LOC645591*	hypothetical protein LOC645591	7q22.1	4.8	30.0	12.0
1561340_at	---	CDNA clone IMAGE:4838684	---	18.3	7.8	11.9
1553153_at	*ATP6V0D2*	ATPase, H+ transporting, lysosomal 38kDa, V0 subunit d2	---	4.8	28.5	11.7
209283_at	*CRYAB*	crystallin, alpha B	11q22.3-23.1	2.8	40.8	10.7
207882_at	*HSAJ2425*	p65 protein	---	3.1	36.1	10.6
203603_s_at	*ZEB2*	zinc finger E-box binding homeobox 2	2q22	3.9	26.5	10.2
1553155_x_at	*ATP6V0D2*	ATPase, H+ transporting, lysosomal 38kDa, V0 subunit d2	---	8.1	11.8	9.7
214505_s_at	*FHL1*	four and a half LIM domains 1	Xq26	8.7	10.5	9.6
221047_s_at	*MARK1*	MAP/microtubule affinity-regulating kinase 1	1q41	16.7	5.2	9.3
1554966_a_at	*FILIP1L*	filamin A interacting protein 1-like	3q12.1	11.8	7.0	9.1
231945_at	*FILIP1*	filamin A interacting protein 1	6q14.1	4.9	16.5	9.0
228982_s_at	*USP42*	Ubiquitin specific peptidase 42	7p22.1	3.3	24.3	8.9
234790_at	---	CDNA: FLJ21261 fis, clone COL01461	1p32.1	5.3	15.0	8.9
224049_at	*KCNK17*	potassium channel, subfamily K, member 17	6p21.1	7.1	9.9	8.4
232523_at	*MEGF10*	multiple EGF-like-domains 10	5q33	2.1	31.7	8.2
228333_at	---	Full length insert cDNA clone YT94E02	---	2.8	22.5	8.0
217139_at	*VDAC1*	voltage-dependent anion channel 1	5q31	5.4	11.8	8.0
210220_at	*FZD2*	frizzled homolog 2 (Drosophila)	17q21.1	2.8	21.5	7.8
227400_at	*NFIX*	nuclear factor I/X (CCAAT-binding transcription factor)	19p13.3	2.0	27.0	7.4
214492_at	*SGCD*	sarcoglycan, delta	5q33-34	2.5	19.8	7.0
1563318_s_at	*MAGIX*	MAGI family member, X-linked	Xp11.23	2.4	20.3	7.0
207755_at	---	---	---	2.1	22.3	6.8
201669_s_at	*MARCKS*	myristoylated alanine-rich protein kinase C substrate	6q22.2	8.2	5.7	6.8
209560_s_at	*DLK1*	delta-like 1 homolog (Drosophila)	14q32	9.5	4.6	6.6
209738_x_at	*PSG6*	pregnancy specific beta-1-glycoprotein 6	19q13.2	3.8	11.2	6.5
228573_at	---	Full-length cDNA clone CS0DD001YA12 of Neuroblastoma	---	2.6	16.1	6.5
240411_at	*FLJ40243*	hypothetical protein FLJ40243	5p13.1	6.5	6.3	6.4
219993_at	*SOX17*	SRY (sex determining region Y)-box 17	8q11.23	2.2	18.4	6.3
212514_x_at	*DDX3X*	DEAD (Asp-Glu-Ala-Asp) box polypeptide 3, X-linked	Xp11.3-11.23	2.3	15.4	6.0
209926_at	*MEF2B*	myocyte enhancer factor 2B	19p12	8.8	4.0	6.0
219778_at	*ZFPM2*	zinc finger protein, multitype 2	8q23	4.6	7.6	5.9
226679_at	*SLC26A11*	solute carrier family 26, member 11	17q25.3	2.2	15.7	5.9
239572_at	*GJA3*	gap junction protein, alpha 3, 46kDa	---	7.7	4.5	5.9
212956_at	*TBC1D9*	TBC1 domain family, member 9	4q31.21	2.6	13.1	5.8
202403_s_at	*COL1A2*	collagen, type I, alpha 2	7q22.1	3.8	8.7	5.8
234351_x_at	*TRPS1*	trichorhinophalangeal syndrome I	8q24.12	2.8	11.5	5.7
212503_s_at	*DIP2C*	DIP2 disco-interacting protein 2 homolog C (Drosophila)	10p15.3	2.1	14.4	5.5

217673_x_at	*GNAS*	GNAS complex locus	20q13.3	2.2	13.8	5.5
232416_at	*BRUNOL5*	bruno-like 5, RNA binding protein (Drosophila)	19p13	8.2	3.6	5.4
207777_s_at	*SP140*	SP140 nuclear body protein	2q37.1	4.5	6.5	5.4
206864_s_at	*HRK*	harakiri, BCL2 interacting protein	12q24.22	2.1	13.8	5.4
224064_s_at	*DHDDS*	dehydrodolichyl diphosphate synthase	1p36.11 /Xq28	6.7	4.3	5.4
1558706_a_at	*ATOH8*	atonal homolog 8 (Drosophila)	2p11.2	6.5	4.4	5.3
231229_at	*HILS1*	histone linker H1 domain, spermatid-specific 1	17q21.33	3.8	7.2	5.2
225897_at	*MARCKS*	myristoylated alanine-rich protein kinase C substrate	---	8.6	3.2	5.2
1556829_at	*TIPARP*	TCDD-inducible poly(ADP-ribose) polymerase	3q25.31	5.5	5,0	5.2
223642_at	*ZIC2*	Zic family member 2	13q32	2.8	9.8	5.2
239673_at	---	Transcribed locus	4q31.1	3.4	8,0	5.2
228141_at	*LOC493869*	Similar to RIKEN cDNA 2310016C16	5q11.2	6.4	3.9	5.0
220245_at	*SLC45A2*	solute carrier family 45, member 2	5p13.3	5.0	4.9	5.0
224340_at	---	---	---	5.3	4.7	5.0
1558999_x_at	*PDPR*	pyruvate dehydrogenase phosphatase regulatory subunit	16q22.1 /q22.3	11.2	2.1	4.9
204506_at	*PPP3R1*	protein phosphatase 3, regulatory subunit B, alpha isoform	2p15	2.6	9.1	4.8
227711_at	*GTSF1*	gametocyte specific factor 1	12q13.2	4.1	5.6	4.8
1554371_at	*PKD1L2*	polycystic kidney disease 1-like 2	16q23.2	4.0	5.9	4.8
221760_at	*MAN1A1*	Mannosidase, alpha, class 1A, member 1	6q22	4.2	5.5	4.8
204069_at	*MEIS1*	Meis homeobox 1	2p14-p13	2.6	8.9	4.8
208523_x_at	*HIST1H2BI*	histone cluster 1, H2bi	6p21.3	4.0	5.8	4.8
205481_at	*ADORA1*	adenosine A1 receptor	1q32.1	7.1	3.2	4.8
235101_at	*FNBP4*	formin binding protein 4	11p11.2	6.6	3.4	4.7
238946_at	---	Transcribed locus	11q21	4.3	5.1	4.7
227108_at	*STARD9*	StAR-related lipid transfer (START) domain containing 9	15q15.2	4.4	5.0	4.7
227812_at	*TNFRSF19*	tumor necrosis factor receptor superfamily, member 19	13q12.11-12.3	2.1	10.4	4.6
242861_at	---	---	---	3.7	5.9	4.6
204469_at	*PTPRZ1*	protein tyrosine phosphatase, receptor-type, Z polypeptide 1	7q31.3	3,0	7.1	4.6
1568830_at	*IRAK3*	Interleukin-1 receptor-associated kinase 3	2q24.2	4.9	4.2	4.6
242828_at	---	---	2q24.3	2.7	7.7	4.6
209423_s_at	*PHF20*	PHD finger protein 20	20q11.22-11.23	2.7	7.6	4.5
220122_at	*MCTP1*	multiple C2 domains, transmembrane 1	5q15	4.0	5.2	4.5
221530_s_at	*BHLHB3*	basic helix-loop-helix domain containing, class B, 3	12p11.23-12.1	3.6	5.7	4.5
1562730_a_at	---	CDNA clone IMAGE:4110098	17q21	2.3	8.8	4.5
211711_s_at	*PTEN*	phosphatase and tensin homolog	10q23.3	3.9	5.2	4.5
209951_s_at	*MAP2K7*	mitogen-activated protein kinase kinase 7	19p13.3-13.2	2.7	7.5	4.5
1569250_at	*ZNF333*	zinc finger protein 333	19p13	7.2	2.7	4.4
212242_at	*TUBA4A*	tubulin, alpha 4a	2q35	6.5	3.0	4.4
217107_at	*LOC442257*	similar to 40S ribosomal protein S4, Y isoform 2	---	4.2	4.5	4.4
219574_at	*MARCH1*	membrane-associated ring finger (C3HC4) 1	4q32.3	2.7	7.0	4.3
211931_s_at	*HNRPA3 /*<br>*HNRPA3P1*	heterogeneous nuclear ribonucleoprotein A3 pseudogene 1 /<br>heterogeneous nuclear ribonucleoprotein A3	10q11.21 /<br>2q31.2 /Xq27.1	2.7	7.0	4.3
201670_s_at	*MARCKS*	myristoylated alanine-rich protein kinase C substrate	6q22.2	6.0	3.1	4.3
233845_at	---	Clone HQ0452 PRO0452	---	3.6	5.0	4.3
201732_s_at	*CLCN3*	chloride channel 3	4q33	2.3	8.0	4.2
204201_s_at	*PTPN13*	protein tyrosine phosphatase, non-receptor type 13	4q21.3	2.3	7.7	4.2
232096_x_at	---	CDNA: FLJ22140 fis, clone HEP20977	3p14.1	4.4	4.0	4.2
231969_at	*STOX2*	storkhead box 2	4q35.1	3.2	5.4	4.2
241955_at	*HECTD1*	HECT domain containing 1	14q12	2.6	6.5	4.1
212805_at	*PRUNE2*	prune homolog 2 (Drosophila)	9q21.13	2.5	6.7	4.1
224203_at	*SUFU*	suppressor of fused homolog (Drosophila)	10q24.32	2.7	6.2	4.1
204271_s_at	*EDNRB*	endothelin receptor type B	13q22	3.8	4.4	4.1
238145_at	---	Transcribed locus	1q44	2.2	7.4	4.1
206306_at	*RYR3*	ryanodine receptor 3	15q14-15	2.7	6.1	4.1
230556_at	*IMMP1L*	IMP1 inner mitochondrial membrane peptidase-like (S. cer)	11p13	2.9	5.5	4.0
225363_at	*PTEN*	phosphatase and tensin homolog	10q23.3	4.3	3.6	4.0
233614_at	---	CDNA FLJ11661 fis, clone HEMBA1004617	3q13.11	3.9	4.0	4.0
205809_s_at	*WASL*	Wiskott-Aldrich syndrome-like	7q31.3	2.2	7.0	3.9
204770_at	*TAP2*	transporter 2, ATP-binding cassette, sub-family B (MDR/TAP)	6p21.3	3.1	4.9	3.9
214561_at	*LILRP2*	leukocyte immunoglobulin-like receptor pseudogene 2	19q13.4	2.2	6.8	3.9
215800_at	*DUOX1*	dual oxidase 1	5q15.3	2.1	6.9	3.8
1555786_s_at	---	---	---	5.4	2.7	3.8
1555021_a_at	*SCARF1*	scavenger receptor class F, member 1	17p13.3	3.2	4.5	3.8
1553845_x_at	*C10orf67*	chromosome 10 open reading frame 67	10p12.2	2.6	5.4	3.8
242486_at	---	Transcribed locus	15q22.31	5.8	2.4	3.8
201101_s_at	*BCLAF1*	BCL2-associated transcription factor 1	6q22-23	2.9	4.8	3.7
1569958_at	---	Homo sapiens, clone IMAGE:4064953, mRNA	---	4.3	3.2	3.7
211712_s_at	*ANXA9*	annexin A9	1q21	6.2	2.2	3.7
230117_at	*VSTM2A*	V-set and transmembrane domain containing 2A	7p11.2	3.4	4.0	3.7
224566_at	*TncRNA*	trophoblast-derived noncoding RNA	11q13.1	4.6	3.0	3.7
203221_at	*TLE1*	transducin-like enhancer of split 1	9q21.32	2.3	5.8	3.7
228715_at	*ZCCHC12*	zinc finger, CCHC domain containing 12	Xq24	2.8	4.7	3.6
213002_at	*MARCKS*	myristoylated alanine-rich protein kinase C substrate	6q22.2	3.9	3.3	3.6
204451_at	*FZD1*	frizzled homolog 1	7q21	2,0	6.3	3.6

223729_at	*CECR2*	cat eye syndrome chromosome region, candidate 2	22q11.2	2.6	4.9	3.5
230259_at	*C10orf125*	chromosome 10 open reading frame 125	10q26.3	2.1	6.0	3.5
204518_s_at	*PPIC*	peptidylprolyl isomerase C	5q23.2	2.7	4.7	3.5
242134_at	---	Transcribed locus	---	3.4	3.7	3.5
209947_at	*UBAP2L*	ubiquitin associated protein 2-like	1q21.3	2.2	5.7	3.5
203167_at	*TIMP2*	TIMP metallopeptidase inhibitor 2	17q25	2.1	5.8	3.5
216556_x_at	---	---	---	2.1	5.7	3.5
208358_s_at	*UGT8*	UDP glycosyltransferase 8	4q26	2.2	5.4	3.5
1561135_at	---	Full length insert cDNA clone YT88G03	2q21.2	2.8	4.1	3.4
1554417_s_at	*APH1A*	anterior pharynx defective 1 homolog A (C. elegans)	1p36.13-q31.3	2.1	5.5	3.4
224565_at	*TncRNA*	trophoblast-derived noncoding RNA	11q13.1	3.1	3.8	3.4
1555908_at	*FAM120A*	family with sequence similarity 120A	9q22.31	2.4	4.8	3.4
243761_at	*PFTK1*	PFTAIRE protein kinase 1	---	3.5	3.2	3.4
223179_at	*YPEL3*	yippee-like 3 (Drosophila)	16p11.2	2.5	4.4	3.3
219102_at	*RCN3*	reticulocalbin 3, EF-hand calcium binding domain	19q13.33	2.2	5.2	3.3
217211_at	*LOC390861*	similar to cytoplasmic beta-actin	---	3,0	3.7	3.3
224140_at	*NPCDR1*	nasopharyngeal carcinoma, down-regulated 1	3p21.1	2.6	4.2	3.3
1558253_x_at	*ZNF587*	zinc finger protein 587	19q13.43	2.9	3.7	3.3
217492_s_at	*LOC731292 / PTEN / PTENP1*	phosphatase and tensin homolog / phosphatase and tensin homolog , pseudogene 1 / sim. Phosphatidylinositol-3,4,5-trisphosphate 3-phosphatase	10q23.3	3.1	3.5	3.3
1564344_at	*ATP9B*	ATPase, class II, type 9B	18q23	2.0	5.3	3.3
204053_x_at	*PTEN*	phosphatase and tensin homolog	10q23.3	3.5	3.1	3.3
236884_at	---	Transcribed locus	---	3.0	3.5	3.3
219779_at	*ZFHX4*	zinc finger homeobox 4	8q21.11	3.4	3.1	3.3
226324_s_at	*IFT172*	intraflagellar transport 172 homolog	2p23.3	2.2	4.7	3.2
210195_s_at	*PSG1*	pregnancy specific beta-1-glycoprotein 1	9q13.2	3.1	3.3	3.2
1555565_s_at	*TAPBP*	TAP binding protein (tapasin)	6p21.3	2.0	5.0	3.2
214657_s_at	*TncRNA*	Trophoblast-derived noncoding RNA	11q13.1	4.6	2.1	3.1
213448_at	---	CDNA FLJ31688 fis, clone NT2RI2005520	1q21	2.6	3.8	3.1
1563772_a_at	*LAMA3*	laminin, alpha 3	18q11.2	2.4	4.1	3.1
230717_at	*LCN12*	lipocalin 12	9q34.3	4.3	2.3	3.1
201401_s_at	*ADRBK1*	adrenergic, beta, receptor kinase 1	11q13	3.4	2.9	3.1
210021_s_at	*CCNO*	cyclin O	5p15.2-13.1	2.5	3.9	3.1
217484_at	*CR1*	complement component (3b/4b) receptor 1	1q32	2.1	4.5	3.1
217009_at	*PGK2*	phosphoglycerate kinase 2	6p12.3	2.4	4.0	3.1
237218_at	---	---	1p22.2	2.1	4.6	3.1
241516_at	---	---	11p14.2	2.1	4.4	3.1
207004_at	*BCL2*	B-cell CLL/lymphoma 2	18q21.33|q21.3	2.4	4.0	3.1
1561726_s_at	---	Homo sapiens, clone IMAGE:4940779, mRNA	11p15.5	2.1	4.4	3.0
1569905_at	*HSD11B1L*	hydroxysteroid (11-beta) dehydrogenase 1-like	19p13.3	2.5	3.7	3.0
228601_at	*LOC401022*	hypothetical LOC401022	2q31.2	2.5	3.6	3.0
202397_at	*NUTF2*	nuclear transport factor 2	16q22.1	2.4	3.7	3.0
229298_at	---	---	---	2.4	3.7	3.0
209970_x_at	*CASP1*	caspase 1, apoptosis-related cysteine peptidase	11q23	3.4	2.6	3.0
43934_at	*GPR137*	G protein-coupled receptor 137	11cen-q22.3	2.2	4.0	3.0
202767_at	*ACP2*	acid phosphatase 2, lysosomal	11p12-p11	2.3	3.8	3.0
222622_at	*LOC283871*	hypothetical protein LOC283871	16p13.3	2.7	3.3	3.0
237187_at	---	Transcribed locus, strongly similar to XP_509406.1	---	2.1	4.2	3.0
239870_at	*SPATS1*	spermatogenesis associated, serine-rich 1	6p21.1	3.3	2.7	2.9
239681_at	*C17orf56*	chromosome 17 open reading frame 56	17q25.3	2.6	3.4	2.9
1570315_at	*LOC283902*	hypothetical gene supported by BC019009	16q22.3	2.5	3.4	2.9
201534_s_at	*UBL3*	ubiquitin-like 3	13q12-q13	3.3	2.6	2.9
209782_s_at	*DBP*	D site of albumin promoter (albumin D-box) binding protein	19q13.3	2.2	3.9	2.9
205347_s_at	*TMSL8*	thymosin-like 8	Xq21.33-22.3	3.3	2.6	2.9
1552734_at	*RICTOR*	rapamycin-insensitive companion of mTOR	5p13.1	2.5	3.3	2.9
209093_s_at	*GBA / GBAP*	glucosidase, beta; acid / glucosidase, beta; acid, pseudogene	1q21	3.2	2.7	2.9
1561258_at	---	---	---	2.7	3.1	2.9
206584_at	*LY96*	lymphocyte antigen 96	8q21.11	3.6	2.3	2.9
203387_s_at	*TBC1D4*	TBC1 domain family, member 4	13q22.2	2.0	4.1	2.9
208588_at	*FKSG2*	apoptosis inhibitor	8p11.2	2.2	3.7	2.9
235285_at	---	Transcribed locus	---	2.2	3.6	2.8
219745_at	*TMEM180*	transmembrane protein 180	10q24.32	2.2	3.7	2.8
242738_s_at	*ZFHX3*	zinc finger homeobox 3	---	2.1	3.8	2.8
243648_at	---	---	22q12-q13	2.2	3.4	2.8
1559514_at	---	CDNA FLJ37869 fis, clone BRSSN2017422	---	2.2	3.4	2.8
201548_s_at	*JARID1B*	jumonji, AT rich interactive domain 1B	1q32.1	2.6	2.9	2.7
217337_at	*LOC646677 / LOC650674*	similar to aconitase 2, mitochondrial	22q12.3	3.1	2.4	2.7
1570567_at	---	CDNA clone IMAGE:4753178	5q13.2	2.8	2.7	2.7
202258_s_at	*RP11-298P3.3*	phosphonoformate immuno-associated protein 5	13q12-13	2.7	2.8	2.7
214449_s_at	*RHOQ*	ras homolog gene family, member Q	2p21	2.3	3.2	2.7
243615_at	---	Transcribed locus	---	2.3	3.3	2.7

1556284_at	*PPA2*	Pyrophosphatase (inorganic) 2	4q25	3.3	2.3	2.7
231756_at	*ZP4*	zona pellucida glycoprotein 4	1q43	2.2	3.3	2.7
201060_x_at	*STOM*	stomatin	9q34.1	2.4	3.1	2.7
243606_at	---	Transcribed locus	---	2.2	3.3	2.7
208089_s_at	*TDRD3*	tudor domain containing 3	13q21.2	3.6	2.0	2.7
228204_at	*PSMB4*	Proteasome (prosome, macropain) subunit, beta type, 4	1q21	2.2	3.3	2.7
34726_at	*CACNB3*	calcium channel, voltage-dependent, beta 3 subunit	12q13	2.5	2.8	2.7
213430_at	*RUFY3*	RUN and FYVE domain containing 3	4q13.3	2.3	3.1	2.7
213844_at	*HOXA5*	homeobox A5	7p15-p14	2.0	3.5	2.7
635_s_at	*PPP2R5B*	protein phosphatase 2, regulatory subunit B', beta isoform	11q12-13	2.1	3.3	2.7
243007_at	---	CDNA FLJ43181 fis, clone FCBBF3016134	10q11.22	2.7	2.5	2.6
202019_s_at	*LANCL1*	LanC lantibiotic synthetase component C-like 1 (bacterial)	2q33-35	3.0	2.3	2.6
222772_at	*MYEF2*	myelin expression factor 2	15q21.1	2.2	3.1	2.6
208485_x_at	*CFLAR*	CASP8 and FADD-like apoptosis regulator	2q33-34	2.2	3.0	2.6
202283_at	*SERPINF1*	serpin peptidase inhibitor, clade F , member 1	17p13.1	3.4	2.0	2.6
222806_s_at	*C11orf30*	chromosome 11 open reading frame 30	11q13.5	2.3	2.9	2.6
225816_at	*PHF17*	PHD finger protein 17	---	2.1	3.2	2.6
1566549_at	---	MRNA; cDNA DKFZp564C1382 (from DKFZp564C1382)	Xp22.33	2.4	2.8	2.6
230481_at	*ACY3*	aspartoacylase (aminocyclase) 3	11q13.2	2.8	2.4	2.6
1563521_at	*MOBKL2B*	MOB1, Mps One Binder kinase activator-like 2B (yeast)	9p21.2	2.8	2.3	2.5
224694_at	*ANTXR1*	anthrax toxin receptor 1	2p13.1	2.4	2.6	2.5
223703_at	*C10orf11*	chromosome 10 open reading frame 11	10q22.2-22.3	2.8	2.3	2.5
1565860_at	---	Full length insert cDNA clone YP87H12	2p15	2.0	3.2	2.5
223930_at	---	---	---	2.4	2.7	2.5
201123_s_at	*EIF5A*	eukaryotic translation initiation factor 5A	17p13-12	2.0	3.1	2.5
204517_at	*PPIC*	peptidylprolyl isomerase C (cyclophilin C)	5q23.2	2.6	2.5	2.5
229908_s_at	*UNKL*	unkempt homolog (Drosophila)-like	---	2.3	2.7	2.5
219952_s_at	*MCOLN1*	mucolipin 1	19p13.3-13.2	2.3	2.7	2.5
211862_x_at	*CFLAR*	CASP8 and FADD-like apoptosis regulator	2q33-34	2.0	3.1	2.5
213300_at	*ATG2A*	ATG2 autophagy related 2 homolog A (S. cerevisiae)	11q13.1	2.2	2.8	2.5
235275_at	*BMP8B*	Bone morphogenetic protein 8b (osteogenic protein 2)	1p34	2.2	2.9	2.5
1554205_s_at	*ICA1L*	islet cell autoantigen 1,69kDa-like	2q33.1	2.3	2.7	2.5
204137_at	*GPR137B*	G protein-coupled receptor 137B	1q42-43	2.5	2.4	2.4
224229_s_at	*AKT3*	v-akt murine thymoma viral oncogene homolog 3	1q43-44	2.3	2.6	2.4
1553157_at	*LHX4*	LIM homeobox 4	1q25.2	2.5	2.3	2.4
221875_x_at	*HLA-F*	major histocompatibility complex, class I, F	6p21.3	2.3	2.5	2.4
212117_at	*RHOQ*	ras homolog gene family, member Q	2p21	2.3	2.5	2.4
212120_at	*RHOQ*	ras homolog gene family, member Q	2q21.1	2.3	2.5	2.4
214073_at	*CTTN*	cortactin	11q13	2.7	2.1	2.4
1555467_a_at	*CUGBP1*	CUG triplet repeat, RNA binding protein 1	11p11	2.3	2.5	2.4
1554310_a_at	*EIF4G3*	eukaryotic translation initiation factor 4 gamma, 3	1p36.12	2.1	2.7	2.4
204262_s_at	*PSEN2*	presenilin 2 (Alzheimer disease 4)	1q31-42	2.5	2.3	2.4
219828_at	*C9orf86*	chromosome 9 open reading frame 86	9q34.3	2.0	2.8	2.4
235799_at	*NSL1*	NSL1, MIND kinetochore complex component, homolog	1q41	2.4	2.2	2.3
202825_at	*SLC25A4*	solute carrier family 25, member 4	15q15.1	2.4	2.3	2.3
201151_s_at	*MBNL1*	muscleblind-like (Drosophila)	3q25	2.3	2.4	2.3
1554047_at	*TXNDC9*	thioredoxin domain containing 9	2q11.2	2.3	2.3	2.3
232676_x_at	*MYEF2*	myelin expression factor 2	15q21.1	2.1	2.6	2.3
91703_at	*EHBP1L1*	EH domain binding protein 1-like 1	17q25.3 /11q13.1	2.0	2.6	2.3
231725_at	*PCDHB2*	protocadherin beta 2	5q31	2.1	2.5	2.3
1554019_s_at	*C6orf182*	chromosome 6 open reading frame 182	6q21	2.4	2.2	2.3
204806_x_at	*HLA-F*	major histocompatibility complex, class I, F	6p21.3	2.2	2.4	2.3
213249_at	*FBXL7*	F-box and leucine-rich repeat protein 7	5p15.1	2.6	2.0	2.3
222575_at	*SETD5*	SET domain containing 5	3p25.3	2.1	2.4	2.3
220760_x_at	*ZNF665*	zinc finger protein 665	19q13.41	2.3	2.3	2.3
220240_s_at	*TMCO3*	transmembrane and coiled-coil domains 3	13q34	2.4	2.1	2.3
208917_x_at	*NADK*	NAD kinase	1p36.33-36.21	2.0	2.5	2.3
228003_at	*RAB30*	RAB30, member RAS oncogene family	11q12-14	2.5	2.1	2.3
242172_at	*MEIS1*	Meis homeobox 1	2p14-p13	2.5	2.0	2.2
243515_at	---	Homo sapiens, clone IMAGE:3641312, mRNA	15q13.3	2.2	2.3	2.2
227099_s_at	*LOC387763*	hypothetical LOC387763	11p11.2	2.4	2.1	2.2
1554509_a_at	*C10orf97*	chromosome 10 open reading frame 97	10p13	2.4	2.1	2.2
204715_at	*PANX1*	pannexin 1	11q21	2.4	2.0	2.2
216331_at	*ITGA7*	integrin, alpha 7	12q13	2.1	2.3	2.2
1552264_a_at	*MAPK1*	mitogen-activated protein kinase 1	22q11.2|q11.21	2.2	2.2	2.2
225798_at	*JAZF1*	JAZF zinc finger 1	7p15.2-15.1	2.1	2.3	2.2
229255_x_at	---	Transcribed locus	---	2.1	2.3	2.2
1562337_at	*OR7D2*	olfactory receptor, family 7, subfamily D, member 2	19p13.2	2.1	2.3	2.2
202052_s_at	*RAI14*	retinoic acid induced 14	5p13.3-p13.2	2.1	2.2	2.2
211089_s_at	*NEK3*	NIMA (never in mitosis gene a)-related kinase 3	13q14.13	2.1	2.2	2.1
204392_at	*CAMK1*	calcium/calmodulin-dependent protein kinase I	3p25.3	2.1	2.2	2.1
239826_at	---	Transcribed locus	18q11.2	2.0	2.2	2.1
201549_x_at	*JARID1B*	jumonji, AT rich interactive domain 1B	1q32.1	2.0	2.2	2.1
219479_at	*KDELC1*	KDEL (Lys-Asp-Glu-Leu) containing 1	13q33	2.0	2.2	2.1

222422_s_at	*NDFIP1*	Nedd4 family interacting protein 1	5q31.3	2.0	2.2	2.1
221551_x_at	*ST6GALNAC4*	ST6 -N-acetylgalactosaminide alpha-2,6-sialyltransferase 4	9q34	2.1	2.0	2.1
226368_at	*CHST11*	Carbohydrate (chondroitin 4) sulfotransferase 11	12q	2.1	2.0	2.1

Down-regulated genes

Probe Set ID	Gene Symbol	Gene Title	Chromosome location	Fold change in PPP treated cells		
				Line2-T500	Line3-T200	Both
203180_at	*ALDH1A3* *	aldehyde dehydrogenase 1 family, member A3	15q26.3	- 19.3	- 66.7	- 35.9
201012_at	*ANXA1**	annexin A1	9q12-21.2	- 4.7	- 94.3	- 21.0
212444_at	---	CDNA clone IMAGE:6025865	---	- 5.4	- 63.5	- 18.5
224341_x_at	*TLR4**	toll-like receptor 4	9q32-33	- 81.4	- 3.5	- 17.0
1555882_at	*SPIN3**	spindlin family, member 3	Xp11.1	- 113.3	- 2.3	- 16.1
240990_at	*RAB5A**	RAB5A, member RAS oncogene family	3p24-22	- 10.5	- 24.5	- 16.1
227697_at	*SOCS3**	suppressor of cytokine signaling 3	17q25.3	- 9.1	- 26.8	- 15.6
237750_at	*XPNPEP3*	X-prolyl aminopeptidase 3, putative	22q13.31-13.33	- 3.5	- 58.1	- 14.2
213953_at	*KRT20*	keratin 20	17q21.2	- 4.5	- 33.2	- 12.2
1552378_s_at	*RDH10*	retinol dehydrogenase 10 (all-trans)	8q21.11	- 11.9	- 12.0	- 12.0
229876_at	*PHKA1*	Phosphorylase kinase, alpha 1 (muscle)	Xq12-13	- 30.4	- 4.6	- 11.9
237210_at	*NFRKB*	nuclear factor related to kappaB binding protein	11q24-25	- 20.5	- 5.6	- 10.7
235056_at	*ETV6*	ets variant gene 6 (TEL oncogene)	12p13	- 3.0	- 37.9	- 10.7
206683_at	*ZNF165*	zinc finger protein 165	6p21.3	- 9.9	- 11.1	- 10.5
238808_at	---	Transcribed locus	---	- 7.0	- 14.7	- 10.2
207992_s_at	*AMPD3*	adenosine monophosphate deaminase (isoform E)	11p15	- 13.1	- 7.8	- 10.1
205199_at	*CA9*	carbonic anhydrase IX	9p13-12	- 14.7	- 5.8	- 9.2
215720_s_at	*NFYA*	nuclear transcription factor Y, alpha	6p21.3	- 5.8	- 14.3	- 9.1
228083_at	*CACNA2D4*	calcium channel, voltage-dependent, alpha 2/delta subunit 4	12p13.33	- 5.2	- 15.4	- 8.9
236835_at	*LOC645431*	hypothetical protein LOC645431	14q23.3	- 21.0	- 3.1	- 8.0
1564676_a_at	---	MRNA; cDNA DKFZp564D123	3q25.31	- 7.4	- 8.3	- 7.8
208425_s_at	*TANC2*	tetratricopeptide repeat, ankyrin repeat and coiled-coil cont 2	7q23.3	- 13.7	- 4.4	- 7.8
209373_at	*MALL*	mal, T-cell differentiation protein-like	2q13	- 6.9	- 8.3	- 7.6
215813_s_at	*PTGS1*	prostaglandin-endoperoxide synthase 1 (prostaglandin G/H synt	9q32-33.3	- 4.5	- 11.9	- 7.3
227974_at	---	Transcribed locus	---	- 17.1	- 3.1	- 7.3
210630_s_at	*RAD52*	RAD52 homolog	12p13-12.2	- 2.3	- 21.1	- 7.0
237571_at	---	Transcribed locus	21q21.2|q21.3	- 3.3	- 13.1	- 6.6
244239_at	---	Full length insert cDNA YN63H06	chr5p15.1	- 18.0	- 2.4	- 6.6
1556580_a_at	---	CDNA FLJ30798 fis, clone FEBRA2001161	---	- 3.4	- 12.5	- 6.5
1555673_at	*LOC728285 / LOC728934*	similar to keratin associated protein 2-4	17q12-21	- 4.4	- 9.4	- 6.5
1556761_at	---	CDNA FLJ30197 fis, clone BRACE2001423	8p23.1	- 5.6	- 7.2	- 6.4
238484_s_at	---	MRNA; clone CD 43T7	---	- 8.7	- 4.7	- 6.4
1559062_at	---	Homo sapiens, clone IMAGE:5730164, mRNA	---	- 3.3	- 12.2	- 6.3
242161_at	---	Transcribed locus	7p12-14	- 3.2	- 12.4	- 6.3
203108_at	*GPRC5A*	G protein-coupled receptor, family C, group 5, member A	12p13-12.3	- 3.8	- 10.1	- 6.2
220945_x_at	*MANSC1*	MANSC domain containing 1	12p13.2	- 4.1	- 9.1	- 6.1
201324_at	*EMP1*	epithelial membrane protein 1	12p12.3	- 2.2	- 16.3	- 6.0
205110_s_at	*FGF13*	fibroblast growth factor 13	Xq26.3	- 3.4	- 10.6	- 6.0
201325_s_at	*EMP1*	epithelial membrane protein 1	12p12.3	- 2.3	- 15.5	- 6.0
1559128_at	*HSDL2*	hydroxysteroid dehydrogenase like 2	9q32	- 2.4	- 14.5	- 5.9
244009_at	---	Transcribed locus	5q23	- 3.6	- 9.5	- 5.9
219263_at	*RNF128*	ring finger protein 128	Xq22.3	- 8.1	- 4.1	- 5.8
242409_at	---	Full-length cDNA clone CS0CAP003YO10 of Thymus	14q24.3	- 12.4	- 2.6	- 5.7
224480_s_at	*MAG1*	lung cancer metastasis-associated protein	4q21.23	- 4.7	- 6.8	- 5.6
204698_at	*ISG20*	interferon stimulated exonuclease gene 20kDa	15q26	- 3.1	- 10.3	- 5.6
1564950_at	---	CDNA clone IMAGE:4052341	8p21.2	- 2.2	- 13.8	- 5.5
240510_at	*NBN*	Nibrin	8q21	- 5.8	- 5.1	- 5.4
202499_s_at	*SLC2A3*	solute carrier family 2, member 3	12p13.3	- 3.6	- 8.1	- 5.4
1568777_at	*EML5*	echinoderm microtubule associated protein like 5	14q31.3	- 8.2	- 3.5	- 5.4
229011_at	---	Transcribed locus	---	- 2.9	- 9.6	- 5.3
230820_at	---	Transcribed locus	---	- 4.2	- 6.6	- 5.3
213428_s_at	*COL6A1*	collagen, type VI, alpha 1	21q22.3	- 5.7	- 4.7	- 5.2
222941_at	*FLJ11850*	hypothetical protein FLJ11850	4q11	- 2.8	- 8.9	- 5.0
218991_at	*HEATR6*	HEAT repeat containing 6	17q23.1	- 2.0	- 12.3	- 5.0
238210_at	---	Transcribed locus	3q22	- 6.3	- 3.9	- 5.0
214698_at	*ROD1*	ROD1 regulator of differentiation 1 (S. pombe)	9q32	- 3.8	- 6.5	- 4.9
212097_at	*CAV1*	caveolin 1, caveolae protein, 22kDa	7q31.1	- 2.6	- 9.2	- 4.9
242434_at	---	CDNA FLJ31093 fis, clone IMR321000161	---	- 3.2	- 7.3	- 4.9
214579_at	*NPAL3*	NIPA-like domain containing 3	1p36.12-35.1	- 10.3	- 2.3	- 4.9
201295_s_at	---	---	17q11.1	- 5.0	- 4.6	- 4.8
236180_at	---	Transcribed locus	12p13.3	- 6.1	- 3.8	- 4.8
1559705_s_at	*PHKA2*	phosphorylase kinase, alpha 2 (liver)	Xp22.2-22.1	- 2.5	- 9.0	- 4.8
208614_s_at	*FLNB*	filamin B, beta (actin binding protein 278)	3p14.3	- 4.6	- 4.9	- 4.7
234049_at	---	Clone PP1195 unknown mRNA	8p23.1	- 2.0	- 10.8	- 4.7
203030_s_at	*PTPRN2*	protein tyrosine phosphatase, receptor type, N pp 2	7q36	- 6.1	- 3.5	- 4.6

1556814_a_at	---	Full length insert cDNA clone ZA96G04	---	- 2.7	- 7.9	- 4.6
213895_at	*EMP1*	epithelial membrane protein 1	12p12.3	- 3.1	- 6.7	- 4.5
202067_s_at	*LDLR*	low density lipoprotein receptor	19p13.3	- 2.2	- 9.0	- 4.5
1561486_at	---	MRNA; cDNA DKFZp666M193	---	- 2.7	- 7.2	- 4.5
224832_at	*DUSP16*	dual specificity phosphatase 16	12p13	- 3.3	- 5.8	- 4.4
204363_at	*F3*	coagulation factor III (thromboplastin, tissue factor)	1p22-21	- 3.8	- 5.0	- 4.4
203821_at	*HBEGF*	heparin-binding EGF-like growth factor	5q23	- 3.0	- 6.5	- 4.4
209765_at	*ADAM19*	ADAM metallopeptidase domain 19 (meltrin beta)	5q32-33	- 5.2	- 3.7	- 4.4
201645_at	*TNC*	tenascin C (hexabrachion)	9q33	- 6.0	- 3.1	- 4.3
216859_x_at	---	---	---	- 5.8	- 3.2	- 4.3
220233_at	*FBXO17*	F-box protein 17	19q13.2	- 3.8	- 4.9	- 4.3
233730_at	*FAM135A*	family with sequence similarity 135, member A	6q12-13	- 4.8	- 3.8	- 4.3
222088_s_at	*SLC2A3*	solute carrier family 2, member 3	12p13.3	- 3.9	- 4.6	- 4.3
220719_at	*FLJ13769*	hypothetical protein FLJ13769	12q24.31	- 3.6	- 5.0	- 4.2
202498_s_at	*SLC2A3*	solute carrier family 2, member 3	12p13.3	- 2.1	- 8.3	- 4.2
204530_s_at	*TOX*	thymocyte selection-associated high mobility group box	8q12.1	- 6.1	- 2.9	- 4.2
208791_at	*CLU*	clusterin	8p21-12	- 4.7	- 3.7	- 4.2
218625_at	*NRN1*	neuritin 1	6p25.1	- 5.0	- 3.5	- 4.2
38037_at	*HBEGF*	heparin-binding EGF-like growth factor	5q23	- 5.5	- 3.2	- 4.2
230757_at	*FLJ44796*	hypothetical FLJ44796	5q31.1	- 3.1	- 5.6	- 4.1
239762_at	*LOC286437*	hypothetical protein LOC286437	Xq22.2	- 5.1	- 3.3	- 4.1
235086_at	*THBS1*	Thrombospondin 1	15q15	- 3.8	- 4.3	- 4.0
209917_s_at	*TP53AP1*	TP53 activated protein 1	7q21.1	- 2.1	- 7.6	- 4.0
1560019_at	*MGC11082*	Hypothetical protein MGC11082	18p11.3	- 2.9	- 5.5	- 4.0
238439_at	*ANKRD22*	ankyrin repeat domain 22	10q23.31	- 6.1	- 2.6	- 4.0
1559528_at	*PCGF3*	Polycomb group ring finger 3	---	- 4.0	- 3.9	- 4.0
227732_at	*ATXN7L1*	ataxin 7-like 1	7q22.1	- 2.1	- 7.4	- 3.9
238813_at	---	Transcribed locus	---	- 4.7	- 3.3	- 3.9
244470_at	*RNF12*	Ring finger protein 12	Xq13-21	- 3.5	- 4.4	- 3.9
1554279_a_at	*CXorf34*	chromosome X open reading frame 34	Xq22.1	- 7.5	- 2.0	- 3.9
234028_at	---	Clone L49 HERV-K-T47-like long terminal repeat sequence	3q27.2	- 3.2	- 4.7	- 3.9
243290_at	*WWC1*	WW and C2 domain containing 1	3p14.1	- 2.5	- 6.0	- 3.9
1557065_at	*YLPM1*	YLP motif containing 1	14q24.3	- 2.1	- 6.9	- 3.8
212473_s_at	*MICAL2*	microtubule associated monoxygenase	11p15.3	- 6.7	- 2.2	- 3.8
203065_s_at	*CAV1*	caveolin 1, caveolae protein, 22kDa	7q31.1	- 2.5	- 5.7	- 3.8
201951_at	*ALCAM*	activated leukocyte cell adhesion molecule	3q13.1	- 5.0	- 2.9	- 3.8
39248_at	*AQP3*	aquaporin 3	9p13	- 2.7	- 5.2	- 3.8
205596_s_at	*SMURF2*	SMAD specific E3 ubiquitin protein ligase 2	17q22-23	- 3.4	- 4.1	- 3.8
1561167_at	---	Full length insert cDNA clone YA75A09	12p13	- 4.3	- 3.3	- 3.8
232020_at	*SMURF2*	SMAD specific E3 ubiquitin protein ligase 2	17q22-23	- 4.0	- 3.5	- 3.7
230127_at	---	Transcribed locus	---	- 5.1	- 2.6	- 3.7
202105_at	*IGBP1*	immunoglobulin (CD79A) binding protein 1	Xq13.1-13.3	- 3.9	- 3.4	- 3.7
237968_at	*ARL6IP2*	ADP-ribosylation factor-like 6 interacting protein 2	2p22.2-22.1	- 3.3	- 4.0	- 3.6
202254_at	*SIPA1L1*	Signal-induced proliferation-associated 1 like 1	14q24.2	- 2.1	- 6.2	- 3.6
215163_at	---	---	---	- 2.7	- 4.8	- 3.6
1559722_at	---	CDNA clone IMAGE:4830182	9q22.32	- 3.4	- 3.9	- 3.6
235494_at	---	CDNA clone IMAGE:5284125	---	- 3.2	- 4.1	- 3.6
234072_at	*SEMA4A*	semaphorin 4A	1q22	- 2.6	- 5.0	- 3.6
210319_x_at	*MSX2*	msh homeobox 2	5q34-35	- 2.4	- 5.2	- 3.6
1556655_s_at	---	CDNA FLJ38740 fis, clone KIDNE2011782	---	- 3.0	- 4.2	- 3.6
232113_at	---	CDNA FLJ38472 fis, clone FEBRA2022148	11q24.1	- 5.4	- 2.3	- 3.6
241272_at	---	Transcribed locus	9q33	- 3.5	- 3.6	- 3.6
219563_at	*C14orf139*	chromosome 14 open reading frame 139	14q32.13	- 2.2	- 5.6	- 3.5
209679_s_at	*LOC57228*	small trans-membrane and glycosylated protein	12q13.13	- 5.2	- 2.3	- 3.5
224964_s_at	*GNG2*	guanine nucleotide binding protein (G protein), gamma 2	14q21	- 2.4	- 4.9	- 3.5
1552727_s_at	*ADAMTS17*	ADAM metallopeptidase with thrombospondin type 1 motif, 17	15q24	- 5.4	- 2.2	- 3.4
211106_at	---	---	6p21.1-21.3	- 3.5	- 3.2	- 3.4
205016_at	*TGFA*	transforming growth factor, alpha	2p13	- 3.7	- 3.0	- 3.4
1565627_a_at	---	Full length insert cDNA clone ZD55G10	15q26.3	- 3.1	- 3.6	- 3.3
1556722_a_at	*FLJ33706*	hypothetical protein FLJ33706	20q11.21	- 2.2	- 4.9	- 3.3
206385_s_at	*ANK3*	ankyrin 3, node of Ranvier (ankyrin G)	10q21	- 5.1	- 2.2	- 3.3
220663_at	*IL1RAPL1*	interleukin 1 receptor accessory protein-like 1	Xp22.1-21.3	- 3.3	- 3.2	- 3.3
208613_s_at	*FLNB*	filamin B, beta	3p14.3	- 2.1	- 5.0	- 3.3
217466_x_at	*RPS2*	ribosomal protein S2	---	- 3.5	- 3.0	- 3.3
226485_at	---	CDNA FLJ37658 fis, clone BRHIP2010593	---	- 2.7	- 3.8	- 3.2
226021_at	*RDH10*	retinol dehydrogenase 10	8q21.11	- 3.0	- 3.4	- 3.2
57540_at	*RBKS*	ribokinase	2p23.3	- 3.8	- 2.7	- 3.2
236716_at	---	CDNA clone IMAGE:5303125	---	- 3.8	- 2.6	- 3.2
225842_at	*PHLDA1*	pleckstrin homology-like domain, family A, member 1	12q15	- 3.3	- 3.1	- 3.2
206076_at	*LRRC23*	leucine rich repeat containing 23	12p13	- 3.1	- 3.2	- 3.1
217999_s_at	*PHLDA1*	pleckstrin homology-like domain, family A, member 1	12q15	- 3.1	- 3.2	- 3.1
1570439_at	---	Clone FLB9835 PRO2660	6q26	- 2.6	- 3.7	- 3.1
232779_at	---	CDNA FLJ20781 fis, clone COL04235	6p25	- 3.0	- 3.2	- 3.1
232413_at	---	CDNA FLJ13497 fis, clone PLACE1004518	10q11	- 3.6	- 2.7	- 3.1

204984_at	*GPC4*	glypican 4	Xq26.1	- 4.4	- 2.2	- 3.1
228005_at	*ZXDB*	zinc finger, X-linked, duplicated B	Xp11.21	- 2.7	- 3.5	- 3.1
227467_at	*RDH10*	retinol dehydrogenase 10 (all-trans)	8q21.11	- 3.0	- 3.2	- 3.1
232919_at	*AFG3L2*	AFG3 ATPase family gene 3-like 2 (yeast)	18p11	- 2.4	- 3.9	- 3.1
218450_at	*HEBP1*	heme binding protein 1	12p13.1	- 2.1	- 4.4	- 3.1
213093_at	*PRKCA*	protein kinase C, alpha	17q22-23.2	- 3.4	- 2.8	- 3.0
242926_at	---	Transcribed locus	2q33.3	- 2.0	- 4.5	- 3.0
201860_s_at	*PLAT*	plasminogen activator, tissue	8p12	- 3.0	- 2.9	- 3.0
202718_at	*IGFBP2*	insulin-like growth factor binding protein 2, 36kDa	2q33-34	- 3.4	- 2.6	- 3.0
232502_at	*FLJ34077 /* *LOC728558*	weakly similar to zinc finger protein 195 / hypothetical protein LOC728558	10q23-24	- 2.1	- 4.1	- 3.0
208792_s_at	*CLU*	clusterin	8p21-12	- 3.6	- 2.4	- 3.0
225171_at	*ARHGAP18*	Rho GTPase activating protein 18	6q22.33	- 2.1	- 4.2	- 3.0
232998_at	---	CDNA FLJ14173 fis, clone NT2RP2002755	4q31.3	- 4.2	- 2.1	- 3.0
209098_s_at	*JAG1*	jagged 1 (Alagille syndrome)	20p12.1-11.23	- 3.0	- 2.9	- 3.0
200637_s_at	*PTPRF*	protein tyrosine phosphatase, receptor type, F	1p34	- 3.5	- 2.5	- 2.9
219222_at	*RBKS*	ribokinase	2p23.3	- 2.6	- 3.3	- 2.9
202609_at	*EPS8*	epidermal growth factor receptor pathway substrate 8	12q13	- 2.1	- 4.0	- 2.9
227372_s_at	*BAIAP2L1*	BAI1-associated protein 2-like 1	7q21.3	- 2.0	- 4.2	- 2.9
1560609_at	*CRYZL1*	crystallin, zeta (quinone reductase)-like 1	21q21.3	- 4.1	- 2.0	- 2.9
236915_at	*LOC441054*	hypothetical protein LOC441054	---	- 3.2	- 2.7	- 2.9
244356_at	---	Transcribed locus	7q11.23	- 2.3	- 3.6	- 2.9
233707_at	---	CDNA FLJ14174 fis, clone NT2RP2002843	3p14.2	- 2.4	- 3.4	- 2.9
1568983_a_at	---	CDNA clone IMAGE:5261717	---	- 3.7	- 2.2	- 2.9
205419_at	*EBI2*	Epstein-Barr virus induced gene 2	13q32.3	- 2.2	- 3.8	- 2.9
211998_at	*H3F3B*	H3 histone, family 3B (H3.3B)	17q25	- 2.5	- 3.3	- 2.9
1559142_at	*MYST3*	MYST histone acetyltransferase 3	8p11	- 2.9	- 2.8	- 2.9
243310_at	---	Transcribed locus	20p13	- 3.7	- 2.2	- 2.9
227205_at	*TAF1*	TAF1 RNA polymerase II	Xq13.1	- 2.5	- 3.3	- 2.8
1553901_x_at	*ZNF486*	zinc finger protein 486	19p12	- 2.8	- 2.8	- 2.8
240042_at	*FIBCD1*	fibrinogen C domain containing 1	9q34.12-34.13	- 2.4	- 3.3	- 2.8
223553_s_at	*DOK3*	docking protein 3	5q35.3	- 3.9	- 2.0	- 2.8
212552_at	*HPCAL1*	hippocalcin-like 1	2p25.1	- 2.1	- 3.7	- 2.8
241360_at	*CCDC15*	coiled-coil domain containing 15	11q24.2	- 2.3	- 3.3	- 2.8
204286_s_at	*PMAIP1*	phorbol-12-myristate-13-acetate-induced protein 1	18q21.32	- 2.5	- 3.1	- 2.8
202085_at	*TJP2*	tight junction protein 2	9q13-q21	- 2.0	- 3.8	- 2.8
201373_at	*PLEC1*	plectin 1, intermediate filament binding protein 500kDa	8q24	- 2.9	- 2.7	- 2.8
235819_at	---	Transcribed locus	---	- 2.1	- 3.7	- 2.8
1558017_s_at	---	CDNA FLJ14942 fis, A-PLACE1011185	12q21	- 3.6	- 2.1	- 2.7
1555681_at	---	MUSP1 (MUSP1)	---	- 2.9	- 2.6	- 2.7
222450_at	*TMEPAI*	transmembrane, prostate androgen induced RNA	20q13.31-13.33	- 3.7	- 2.0	- 2.7
1568623_a_at	*SLC35E4*	solute carrier family 35, member E4	22q12.2	- 2.7	- 2.8	- 2.7
239760_at	---	CDNA FLJ30257 fis, clone BRACE2002467	17q22	- 2.4	- 3.1	- 2.7
238462_at	*UBASH3B*	ubiquitin associated and SH3 domain containing, B	11q24.1	- 2.5	- 2.9	- 2.7
229864_at	*TMEM103*	Transmembrane protein 103	3p21.31	- 3.0	- 2.4	- 2.7
232406_at	---	CDNA FLJ13731 fis, clone PLACE3000142	20p12.1-11.23	- 3.1	- 2.3	- 2.7
238909_at	*S100A10*	S100 calcium binding protein A10	1q21	- 2.4	- 2.9	- 2.7
232362_at	*CCDC18*	coiled-coil domain containing 18	1p22.1	- 2.4	- 3.0	- 2.7
1556608_a_at	*EHD4*	EH-domain containing 4	15q11.1	- 3.1	- 2.3	- 2.7
236118_at	*LOC728473*	hypothetical protein LOC728473	---	- 2.3	- 3.1	- 2.7
212838_at	*DNMBP*	dynamin binding protein	10q24.2	- 3.2	- 2.2	- 2.6
230206_at	*DOCK5*	Dedicator of cytokinesis 5	8p21.2	- 2.8	- 2.5	- 2.6
238669_at	*PTGS1*	prostaglandin-endoperoxide synthase 1	9q32-33.3	- 3.0	- 2.3	- 2.6
1554122_a_at	*HSD17B12*	hydroxysteroid (17-beta) dehydrogenase 12	11p11.2	- 2.0	- 3.4	- 2.6
206583_at	*ZNF673*	zinc finger protein 673	Xp11.3	- 2.3	- 3.0	- 2.6
210128_s_at	*LTB4R*	leukotriene B4 receptor	14q11.2-12	- 3.1	- 2.2	- 2.6
225029_at	*LOC550643*	hypothetical protein LOC550643	Xp11.1	- 3.1	- 2.2	- 2.6
222421_at	*UBE2H*	ubiquitin-conjugating enzyme E2H	7q32	- 2.3	- 3.0	- 2.6
209672_s_at	*FLJ20323*	hypothetical protein FLJ20323	7p22-p21	- 2.6	- 2.6	- 2.6
201189_s_at	*ITPR3*	inositol 1,4,5-triphosphate receptor, type 3	6p21	- 2.4	- 2.8	- 2.6
225728_at	*SORBS2*	sorbin and SH3 domain containing 2	4q35.1	- 2.6	- 2.6	- 2.6
235406_x_at	---	CDNA clone IMAGE:4814828	---	- 3.2	- 2.1	- 2.6
202912_at	*ADM*	adrenomedullin	11p15.4	- 2.5	- 2.7	- 2.6
244046_at	*URG4*	up-regulated gene 4	7p13	- 2.2	- 3.1	- 2.6
220149_at	*C2orf54*	chromosome 2 open reading frame 54	2q37.3	- 2.3	- 2.9	- 2.6
225361_x_at	*FAM122B*	family with sequence similarity 122B	Xq26.3	- 2.4	- 2.8	- 2.6
224797_at	*ARRDC3*	arrestin domain containing 3	5q14.3	- 2.7	- 2.5	- 2.6
238587_at	*UBASH3B*	ubiquitin associated and SH3 domain containing, B	11q24.1	- 2.5	- 2.6	- 2.6
218000_s_at	*PHLDA1*	pleckstrin homology-like domain, family A, member 1	12q15	- 2.5	- 2.6	- 2.6
1558447_at	---	CDNA FLJ35687 fis, clone SPLEN2019349	19p13.3	- 2.9	- 2.3	- 2.6
221480_at	*HNRPD*	heterogeneous nuclear ribonucleoprotein D	4q21.1-21.2	- 2.7	- 2.5	- 2.6
242273_at	---	Transcribed locus	---	- 2.1	- 3.1	- 2.6
215195_at	*PRKCA*	protein kinase C, alpha	17q22-23.2	- 2.4	- 2.7	- 2.5
222673_x_at	*FAM122B /*	transmembrane protein 57 /	1p36.11	- 2.7	- 2.3	- 2.5

	TMEM57	family with sequence similarity 122B	Xq26.3			
1565849_a_at	*ZNF576*	Zinc finger protein 576	19q13.31	- 2.4	- 2.7	- 2.5
209008_x_at	*KRT8*	keratin 8	12q13	- 2.7	- 2.4	- 2.5
205462_s_at	*HPCAL1*	hippocalcin-like 1	2p25.1	- 2.9	- 2.2	- 2.5
1553708_at	*MGC16075*	hypothetical protein MGC16075	chr7p12.3	- 2.3	- 2.7	- 2.5
211445_x_at	*NACAP1*	nascent-polypeptide-associated complex α pp pseudogene 1	8q22.3 / 4q32.3	- 3.0	- 2.1	- 2.5
201906_s_at	*CTDSPL*	CTD small phosphatase-like	3p21.3	- 2.1	- 2.9	- 2.5
219580_s_at	*TMC5*	transmembrane channel-like 5	16p12.3	- 2.7	- 2.2	- 2.5
204268_at	*S100A2*	S100 calcium binding protein A2	1q21	- 2.8	- 2.1	- 2.5
220590_at	*ITFG2*	integrin alpha FG-GAP repeat containing 2	12p13.33	- 2.9	- 2.1	- 2.5
1552362_a_at	*LEAP2*	liver expressed antimicrobial peptide 2	5q31.1	- 2.5	- 2.4	- 2.4
216268_s_at	*JAG1*	jagged 1	20p12.1-11.23	- 2.3	- 2.5	- 2.4
202071_at	*SDC4*	syndecan 4	20q12	- 2.1	- 2.9	- 2.4
220172_at	*C2orf37*	chromosome 2 open reading frame 37	2q31.1	- 2.3	- 2.5	- 2.4
233543_s_at	*CCDC98*	coiled-coil domain containing 98	4q21.21-21.23	- 2.1	- 2.7	- 2.4
231183_s_at	*JAG1*	Jagged 1	20p12.1-11.23	- 2.7	- 2.1	- 2.4
205547_s_at	*TAGLN*	transgelin	11q23.2	- 2.4	- 2.4	- 2.4
239944_at	*NAIP*	NLR family, apoptosis inhibitory protein	5q13	- 2.8	- 2.1	- 2.4
236114_at	---	Transcribed locus	21q22.3	- 2.3	- 2.5	- 2.4
220890_s_at	*DDX47*	DEAD box polypeptide 47	12p13.1	- 2.8	- 2.1	- 2.4
240315_at	---	Transcribed locus	6q24.1	- 2.3	- 2.5	- 2.4
201312_s_at	*SH3BGRL*	SH3 domain binding glutamic acid-rich protein like	Xq13.3	- 2.5	- 2.3	- 2.4
238542_at	*ULBP2*	UL16 binding protein 2	6q25	- 2.1	- 2.7	- 2.4
229559_at	*FLJ40125*	hypothetical protein FLJ40125	19q13.32	- 2.6	- 2.1	- 2.4
1563461_at	---	MRNA; cDNA DKFZp686P153	15q25.1	- 2.1	- 2.7	- 2.4
203282_at	*GBE1*	glucan (1,4-alpha-), branching enzyme 1	3p12.3	- 2.0	- 2.7	- 2.4
236526_x_at	*FLJ13611*	hypothetical protein FLJ13611	5q12.3	- 2.2	- 2.6	- 2.4
232612_s_at	*ATG16L1*	ATG16 autophagy related 16-like 1	2q37.1	- 2.2	- 2.4	- 2.3
233778_at	---	Uncharacterized gastric protein ZG16P	10q24.32	- 2.1	- 2.6	- 2.3
1560199_x_at	*FLJ11903*	similar to hypothetical protein MGC40405	5q12.1	- 2.1	- 2.6	- 2.3
203790_s_at	*HRSP12*	heat-responsive protein 12	8q22	- 2.1	- 2.5	- 2.3
202468_s_at	*CTNNAL1*	catenin (cadherin-associated protein), alpha-like 1	9q31.2	- 2.4	- 2.2	- 2.3
230207_s_at	*DOCK5*	Dedicator of cytokinesis 5	8p21.2	- 2.6	- 2.0	- 2.3
239866_at	---	CDNA FLJ14392 fis, clone HEMBA1003166	---	- 2.6	- 2.1	- 2.3
239207_at	*JARID1C*	jumonji, AT rich interactive domain 1C	Xp11.22-11.21	- 2.2	- 2.3	- 2.3
201188_s_at	*ITPR3*	inositol 1,4,5-triphosphate receptor, type 3	6p21	- 2.1	- 2.4	- 2.3
215066_at	*PTPRF*	protein tyrosine phosphatase, receptor type, F	1p34	- 2.4	- 2.2	- 2.3
233506_at	---	Full length insert cDNA clone ZB81B12	---	- 2.2	- 2.3	- 2.3
239897_at	*BCLAF1*	BCL2-associated transcription factor 1	6q22-q23	- 2.2	- 2.3	- 2.3
210653_s_at	*BCKDHB*	branched chain keto acid dehydrogenase E1, beta pp	6q13-q15	- 2.6	- 2.0	- 2.3
33304_at	*ISG20*	interferon stimulated exonuclease gene 20kDa	15q26	- 2.2	- 2.3	- 2.2
200737_at	*PGK1*	phosphoglycerate kinase 1	Xq13	- 2.4	- 2.1	- 2.2
232002_at	---	MRNA; cDNA DKFZp547I084 (	19q13.1	- 2.0	- 2.4	- 2.2
231899_at	*ZC3H12C*	zinc finger CCCH-type containing 12C	11q22.3	- 2.1	- 2.3	- 2.2
222714_s_at	*LACTB2*	lactamase, beta 2	8p22-q22.3	- 2.4	- 2.0	- 2.2
240800_x_at	---	Transcribed locus	7q11.21-11.22	- 2.3	- 2.1	- 2.2
209566_at	*INSIG2*	insulin induced gene 2	2q14.1	- 2.1	- 2.3	- 2.2
243159_x_at	---	Transcribed locus	1q32	- 2.3	- 2.1	- 2.2
209386_at	*TM4SF1*	transmembrane 4 L six family member 1	3q21-25	- 2.3	- 2.1	- 2.2
231886_at	*DKFZP-434B2016*	similar to hypothetical protein LOC284701	7p22.3 /11p15.5 /1p36.33 / 3q29	- 2.2	- 2.1	- 2.2
203431_s_at	*RICS*	Rho GTPase-activating protein	11q24-25	- 2.0	- 2.3	- 2.2
202959_at	*MUT*	methylmalonyl Coenzyme A mutase	6p21	- 2.2	- 2.1	- 2.1
236571_at	---	Transcribed locus	12p13.3	- 2.0	- 2.2	- 2.1
216862_s_at	*MTCP1*	mature T-cell proliferation 1	Xq28	- 2.2	- 2.1	- 2.1
202733_at	*P4HA2*	procollagen-proline, 2-oxoglutarate 4-dioxygenase, α pp II	5q31	- 2.2	- 2.1	- 2.1
200796_s_at	*MCL1*	myeloid cell leukemia sequence 1 (BCL2-related)	1q21	- 2.2	- 2.1	- 2.1
1556110_at	---	CDNA FLJ39819 fis, clone SPLEN2010534	---	- 2.2	- 2.0	- 2.1
201955_at	*CCNC*	cyclin C	6q21	- 2.1	- 2.1	- 2.1
219833_s_at	*EFHC1*	EF-hand domain (C-terminal) containing 1	6p12.3	- 2.0	- 2.2	- 2.1
202331_at	*BCKDHA*	branched chain keto acid dehydrogenase E1, alpha pp	9q13.1-13.2	- 2.1	- 2.0	- 2.1
224833_at	*ETS1*	v-ets erythroblastosis virus E26 oncogene homolog 1	11q23.3	- 2.1	- 2.1	- 2.1
209122_at	*ADFP*	adipose differentiation-related protein	9p22.1	- 2.0	- 2.1	- 2.1
227290_at	---	CDNA FLJ13598 fis, clone PLACE1009921	---	- 2.0	- 2.0	- 2.0

* indicates genes validated by siRNA
