# Supplementary material for: Molecular Characterization of Acquired Tolerance of Tumor Cells to Picropodophyllin (PPP)
Source: PLoS One. 2011 Mar 14;6(3):e14757. doi: 10.1371/journal.pone.0014757 (PMC3056661; doi:10.1371/journal.pone.0014757)
Supplement: Table S5 — Gene list H, up and down-regulated genes in PPP tolerant Line2-T500 and line3-T200 cells (including genes with >2-fold difference vs parental cells). (0.03 MB PDF) [file pone.0014757.s007.pdf]

Table S5. Gene list H, up and down-regulated genes in PPP tolerant Line2-T500 and line3-T200 cells (including genes with >2-fold difference vs parental cells)

Affymetrix Probe Set ID	Gene Symbol	Gene Title	Chromosomal Location
Upregulated genes			
204517_at	*PPIC*	peptidylprolyl isomerase C (cyclophilin C)	5q23.2
225351_at	*FAM45A*	family with sequence similarity 45, member A	10q25
201732_s_at	*CLCN3*	chloride channel 3	4q33
210220_at	*FZD2*	frizzled homolog 2 (Drosophila)	17q21.1
225897_at	*MARCKS*	myristoylated alanine-rich protein kinase C substrate	6q22.2
202258_s_at	*N4BP2L2*	NEDD4 binding protein 2-like 2	13q13.1
226820_at	*ZNF362*	zinc finger protein 362	1p35.1
227812_at	*TNFRSF19*	tumor necrosis factor receptor superfamily, member 19	13q12.11-12.3
223170_at	*TMEM98*	transmembrane protein 98	17q11.2
220122_at	*MCTP1*	multiple C2 domains, transmembrane 1	5q15
203387_s_at	*TBC1D4*	TBC1 domain family, member 4	13q22.2
36564_at	*RNF19B*	ring finger protein 19B	1p35.1
230259_at	*C10orf125*	chromosome 10 open reading frame 125	10q26.3
223412_at	*KBTBD7*	kelch repeat and BTB (POZ) domain containing 7	13q14.11
214492_at	*SGCD*	sarcoglycan, delta	5q33-34
212805_at	*PRUNE2*	prune homolog 2 (Drosophila)	9q21.13
212956_at	*TBC1D9*	TBC1 domain family, member 9	4q31.21
227711_at	*GTSF1*	gametocyte specific factor 1	12q13.2
241771_at	*RIMBP2*	RIMS binding protein 2	12q24.33
201548_s_at	*KDM5B*	lysine (K)-specific demethylase 5B	1q32.1
219102_at	*RCN3*	reticulocalbin 3, EF-hand calcium binding domain	19q13.33
214963_at	*NUP160*	nucleoporin 160kDa	11p11.2
91703_at	*EHBP1L1*	EH domain binding protein 1-like 1	11q13.1
204451_at	*FZD1*	frizzled homolog 1 (Drosophila)	7q21
1570315_at	*HTA*	hepatoma associated protein	16q22.3
243606_at	*FAM55C*	family with sequence similarity 55, member C	3q12.3
231725_at	*PCDHB2*	protocadherin beta 2	5q31
209738_x_at	*PSG6*	pregnancy specific beta-1-glycoprotein 6	19q13.2
231969_at	*STOX2*	storkhead box 2	4q35.1
223642_at	*ZIC2*	Zic family member 2 (odd-paired homolog, Drosophila)	13q32
207777_s_at	*SP140*	SP140 nuclear body protein	2q37.1
232060_at	*ROR1*	receptor tyrosine kinase-like orphan receptor 1	1p32-p31
1554966_a_at	*FILIP1L*	filamin A interacting protein 1-like	3q12.1
206382_s_at	*BDNF*	brain-derived neurotrophic factor	11p13
202403_s_at	*COL1A2*	collagen, type I, alpha 2	7q22.1
209423_s_at	*PHF20*	PHD finger protein 20	20q11.22-11.23
209947_at	*UBAP2L*	ubiquitin associated protein 2-like	1q21.3
241700_at	*ZFHX4*	zinc finger homeobox 4	8q21.11
1553153_at	*ATP6V0D2*	ATPase, H+ transporting, lysosomal 38kDa, V0 subunit d2	---
211204_at	*ME1*	malic enzyme 1, NADP(+)-dependent, cytosolic	6q12
215505_s_at	*STRN3*	striatin, calmodulin binding protein 3	14q13-21
221760_at	*MAN1A1*	Mannosidase, alpha, class 1A, member 1	6q22
201101_s_at	*BCLAF1*	BCL2-associated transcription factor 1	6q22-q23
203577_at	*GTF2H4*	general transcription factor IIH, polypeptide 4, 52kDa	6p21.3
225524_at	*ANTXR2*	anthrax toxin receptor 2	4q21.21
209807_s_at	*NFIX*	nuclear factor I/X (CCAAT-binding transcription factor)	19p13.3
224566_at	*NCRNA00084*	non-protein coding RNA 84	11q13.1
220245_at	*SLC45A2*	solute carrier family 45, member 2	5p13.3
214073_at	*CTTN*	cortactin	11q13
212120_at	*RHOQ*	ras homolog gene family, member Q	2p21
225363_at	*PTEN*	phosphatase and tensin homolog	10q23.3
212514_x_at	*DDX3X*	DEAD (Asp-Glu-Ala-Asp) box polypeptide 3, X-linked	Xp11.3-11.23
206858_s_at	*HOXC6*	homeobox C6	12q13.3
209357_at	*CITED2*	Cbp/p300-interacting transactivator 2	6q23.3

217114_at	*LOC652147*	similar to U5 snRNP-specific protein, 200 kDa	---
211931_s_at	*HNRNPA3*	heterogeneous nuclear ribonucleoprotein A3	10q11.21 /2q31.2
224344_at	*COX6A1*	cytochrome c oxidase subunit VIa polypeptide 1	12q24.2

Down-regulated genes

201087_at	*PXN*	paxillin	12q24.31
222421_at	*UBE2H*	ubiquitin-conjugating enzyme E2H (UBC8 homolog, yeast)	7q32
207643_s_at	*TNFRSF1A*	tumor necrosis factor receptor superfamily, member 1A	12p13.2
238462_at	*UBASH3B*	ubiquitin associated and SH3 domain containing, B	11q24.1
224480_s_at	*AGPAT9*	1-acylglycerol-3-phosphate O-acyltransferase 9	4q21.23
203108_at	*GPRC5A*	G protein-coupled receptor, family C, group 5, member A	12p13-12.3
216005_at	*TNC*	Tenascin C	9q33
225171_at	*ARHGAP18*	Rho GTPase activating protein 18	6q22.33
227372_s_at	*BAIAP2L1*	BAI1-associated protein 2-like 1	7q21.3
212341_at	*YIPF6*	Yip1 domain family, member 6	Xq12
202105_at	*IGBP1*	immunoglobulin (CD79A) binding protein 1	Xq13.1-13.3
209765_at	*ADAM19*	ADAM metallopeptidase domain 19 (meltrin beta)	5q32-33
201887_at	*IL13RA1*	interleukin 13 receptor, alpha 1	Xq24
213428_s_at	*COL6A1*	collagen, type VI, alpha 1	21q22.3
201189_s_at	*ITPR3*	inositol 1,4,5-triphosphate receptor, type 3	6p21
212838_at	*DNMBP*	dynamin binding protein	10q24.2
235692_at	*SH3KBP1*	SH3-domain kinase binding protein 1	Xp22.1-21.3
238909_at	*S100A10*	S100 calcium binding protein A10	1q21
204286_s_at	*PMAIP1*	phorbol-12-myristate-13-acetate-induced protein 1	18q21.32
219833_s_at	*EFHC1*	EF-hand domain (C-terminal) containing 1	6p12.3
225728_at	*SORBS2*	sorbin and SH3 domain containing 2	4q35.1
228574_at	*TMTC2*	Transmembrane and tetratricopeptide repeat containing 2	12q21.31
1560019_at	*MGC11082*	hypothetical LOC84777	18p11.31
224964_s_at	*GNG2*	guanine nucleotide binding protein (G protein), gamma 2	14q21
232020_at	*SMURF2*	SMAD specific E3 ubiquitin protein ligase 2	17q22-23
1555673_at	*KRTAP2-4*	keratin associated protein 2-4	17q12-21
216862_s_at	*MTCP1NB*	mature T-cell proliferation 1 neighbor	Xq28
216971_s_at	*PLEC1*	plectin 1, intermediate filament binding protein 500kDa	8q24
227205_at	*TAF1*	TAF1 RNA polymerase II	Xq13.1
230206_at	*DOCK5*	Dedicator of cytokinesis 5	8p21.2
201373_at	*PLEC1*	plectin 1, intermediate filament binding protein 500kDa	8q24
203821_at	*HBEGF*	heparin-binding EGF-like growth factor	5q23
1552334_at	*TRIOBP*	TRIO and F-actin binding protein	22q13.1
222815_at	*RLIM*	ring finger protein, LIM domain interacting	Xq13-21
228005_at	*ZXDB*	zinc finger, X-linked, duplicated B	Xp11.21
213280_at	*GARNL4*	GTPase activating Rap/RanGAP domain-like 4	17p13.3
232612_s_at	*ATG16L1*	ATG16 autophagy related 16-like 1 (S. cerevisiae)	2q37.1
229785_at	*KRIT1*	KRIT1, ankyrin repeat containing	7q21-22
204894_s_at	*AOC3*	amine oxidase, copper containing 3	17q21
201951_at	*ALCAM*	activated leukocyte cell adhesion molecule	3q13.1
203180_at	*ALDH1A3*	aldehyde dehydrogenase 1 family, member A3	15q26.3
229876_at	*PHKA1*	phosphorylase kinase, alpha 1 (muscle)	Xq12-13
235056_at	*ETV6*	ets variant 6	12p13
206683_at	*ZNF165*	zinc finger protein 165	6p21.3
1555882_at	*SPIN3*	spindlin family, member 3	Xp11.1
213424_at	*KIAA0895*	KIAA0895	7p14.2
1552362_a_at	*LEAP2*	liver expressed antimicrobial peptide 2	5q31.1
238813_at	*ALAS2*	aminolevulinate, delta-, synthase 2	Xp11.21
208791_at	*CLU*	clusterin	8p21-12
202512_s_at	*ATG5*	ATG5 autophagy related 5 homolog (S. cerevisiae)	6q21
238542_at	*ULBP2*	UL16 binding protein 2	6q25
213326_at	*VAMP1*	vesicle-associated membrane protein 1 (synaptobrevin 1)	12p
206385_s_at	*ANK3*	ankyrin 3, node of Ranvier (ankyrin G)	10q21
219911_s_at	*LOC100134295*	similar to Solute carrier organic anion transporter family, 4A1	20q13.33
209679_s_at	*SMAGP*	small trans-membrane and glycosylated protein	12q13.13

213093_at	*PRKCA*	protein kinase C, alpha	17q22-23.2
209373_at	*MALL*	mal, T-cell differentiation protein-like	2q13
39248_at	*AQP3*	aquaporin 3	9p13
202085_at	*TJP2*	tight junction protein 2	9q13-21
226021_at	*RDH10*	retinol dehydrogenase 10	8q21.11
205199_at	*CA9*	carbonic anhydrase IX	9p13-12
224825_at	*DNTTIP1*	deoxynucleotidyltransferase, terminal, interacting protein 1	20q13.12
204268_at	*S100A2*	S100 calcium binding protein A2	1q21
203323_at	*CAV2*	caveolin 2	7q31.1
222088_s_at	*SLC2A14*	solute carrier family 2, member 14	12p13.3 /13.31
211998_at	*H3F3B*	H3 histone, family 3B (H3.3B)	17q25
208614_s_at	*FLNB*	filamin B, beta	3p14.3
201012_at	*ANXA1*	annexin A1	9q12-21.2|
208698_s_at	*NONO*	non-POU domain containing, octamer-binding	Xq13.1
221847_at	*LOC100129361*	hypothetical protein LOC100129361	12p13.2
222450_at	*PMEPA1*	prostate transmembrane protein, androgen induced 1	20q13.31-13.33
208937_s_at	*ID1*	inhibitor of DNA binding 1, dominant negative helix-loop-helix prot	20q11
202499_s_at	*SLC2A3*	solute carrier family 2, member 3	12p13.3
212097_at	*CAV1*	caveolin 1, caveolae protein, 22kDa	7q31.1
216268_s_at	*JAG1*	jagged 1 (Alagille syndrome)	20p12.1-11.23
222673_x_at	*FAM122B*	family with sequence similarity 122B	Xq26.3
201324_at	*EMP1*	epithelial membrane protein 1	12p12.3
202609_at	*EPS8*	epidermal growth factor receptor pathway substrate 8	12q13
219263_at	*RNF128*	ring finger protein 128	Xq22.3
225842_at	*PHLDA1*	pleckstrin homology-like domain, family A, member 1	12q15
212552_at	*HPCAL1*	hippocalcin-like 1	2p25.1
