# Supplementary material for: Molecular Characterization of Acquired Tolerance of Tumor Cells to Picropodophyllin (PPP)
Source: PLoS One. 2011 Mar 14;6(3):e14757. doi: 10.1371/journal.pone.0014757 (PMC3056661; doi:10.1371/journal.pone.0014757)
Supplement: Table S6 — Ontological categories generated from common up-regulated and down-regulated genes in PPP tolerant Line2-T500 and Line3-T200 cells (by DAVID, from gene list U). (0.04 MB PDF) [file pone.0014757.s008.pdf]

Table S6. Ontological categories generated from common up-regulated and down-regulated genes in PPP tolerant Line2-T500 and Line3-T200 cells (by DAVID, from gene list U).

ID	Name of GO category	No. of genes	p-value
UP-REGULATED GENES			
Biological processes			
GO:0008219	cell death	18	0.016
Cellular components			
GO:0044424	intracellular part	134	0.005
GO:0005622	intracellular	138	0.020
GO:0005737	cytoplasm	88	0.021
GO:0043229	intracellular organelle	110	0.048
GO:0044431	Golgi apparatus part	11	0.011
GO:0000139	Golgi membrane	9	0.038
KEGG pathway			
hsa04510:	focal adhesion	8	0.028
SMART			
SM00165	UBA	4	0.006
SP COMMENT			
similarity:	contains 1 UBA domain.	4	0.003
cofactor	magnesium	7	0.039
INTERPRO			
IPR000449	Ubiquitin-associated/translation elongation factor EF1B, N-terminal	4	0.013
IPR015880	Zinc finger, C2H2-like	10	0.032
IPR011992	EF-Hand type	8	0.018
SP PIR KEYWORDS			
Keyword	apoptosis	9	0.020
UP SEQ FEATURE			
domain	UBA	4	0.004
DOWN-REGULATED GENES			
Biological processes			
GO:0008219	cell death	19	0.002
GO:0048522	positive regulation of cellular process	21	0.002
GO:0030154	cell differentiation	32	0.002
GO:0043067	regulation of programmed cell death	14	0.003
GO:0048518	positive regulation of metabolic process	22	0.004
GO:0045595	regulation of cell differentiation	6	0.023
GO:0051094	positive regulation of developmental process	4	0.036
GO:0009611	response to wounding	11	0.010
Molecular functions			
GO:0051213	dioxygenase activity	4	0.020
GO:0016701	oxidoreductase activity, acting on single donors with incorporation of molecular oxygen	4	0.021
Cellular components			
GO:0005622	intracellular	119	0.042
KEGG Pathway			
hsa00280	valine, leucine and isoleucine degradation	4	0.025
INTERPRO			
IPR000742	EGF-like, type 3	9	0.001

IPR006210	EGF	8	0.007
IPR013032	EGF-like region	9	0.015
IPR001452	Src homology-3	8	0.007
IPR001715	Calponin-like actin-binding	4	0.031
SP PIR KEYWORDS			
Key word	egf-like domain	9	0.002
Key word	apoptosis	9	0.009
Key word	SH3 domain	7	0.017
Key word	tyrosine-specific phosphatase	3	0.036
Key word	metalloprotease	5	0.049
Key word	DNA binding	8	0.036
UP SEQ FEATURE			
domain	EGF-like	5	0.006
domain	EGF-like 3	4	0.023
active site	Phosphocysteine intermediate	4	0.044
metal ion-binding site	Zinc (catalytic)	5	0.031
**SP COMMENT**			
similarity	Contains 1 EGF-like domain	5	0.007
similarity	Contains 1 SH3 domain	6	0.042
catalytic activity	Protein tyrosine phosphate + H(2)O = protein tyrosine + phosphate	4	0.050
subcellular location	Membrane; Single-pass type I membrane protein	11	0.017
SMART			
SM00181	EGF	8	0.009
SM00326	SH3	8	0.009
SM00033	CH	4	0.036
