# Supplementary material for: Molecular Characterization of Acquired Tolerance of Tumor Cells to Picropodophyllin (PPP)
Source: PLoS One. 2011 Mar 14;6(3):e14757. doi: 10.1371/journal.pone.0014757 (PMC3056661; doi:10.1371/journal.pone.0014757)
Supplement: Table S7 — Ontological categories generated from common up- and down-regulated genes in PPP tolerant Line2-T500 and Line3-T200 cells (by DAVID, from gene list H). (0.01 MB PDF) [file pone.0014757.s009.pdf]

Table S7. Ontological categories generated from common up- and down-regulated genes in PPP tolerant Line2-T500 and Line3-T200 cells (by DAVID, from gene list H).

ID	Name of category	No. of genes	p-value
UP-REGULATED GENES			
Biological processes			
GO:0002009	morphogenesis of an epithelium	3	0.020
Molecular functions			
GO:0003677	DNA binding	12	0.040
INTERPRO			
IPR001965	Zinc finger, PHD-type	3	0.027
SMART			
SM00249	PHD	3	0.045
DOWN-REGULATED GENES			
Biological processes			
GO:0030154	cell differentiation	18	0.004
GO:0008219	cell death	11	0.005
GO:0006928	cell motility	7	0.014
GO:0016477	cell migration	5	0.038
GO:0003013	circulatory system process	4	0.044
GO:0048523	negative regulation of cellular process	11	0.038
GO:0048519	negative regulation of biological process	11	0.049
Molecular functions			
GO:0004842	ubiquitin-protein ligase activity	4	0.019
GO:0016879	ligase activity, forming carbon-nitrogen bonds	4	0.048
Cellular components			
GO:0044433	cytoplasmic vesicle part	4	0.012
GO:0012506	vesicle membrane	4	0.017
GO:0044459	plasma membrane part	17	0.010
GO:0005886	plasma membrane	23	0.023
GO:0044425	membrane part	36	0.026
INTERPRO			
IPR001452	Src homology-3	7	<0.001
SMART			
SM00326	SH3	7	<0.001
SP PIR KEYWORDS			
Key word	SH3 domain	6	0.002
Key word	EF hand	3	0.024
Key word	membrane	35	0.006
Key word	cytoskeleton	6	0.019
